# Trehalose-Releasing Nanogels: Study on Trehalose Release and Insights into Selected Biologically Relevant Aspects

**DOI:** 10.1021/acs.biomac.4c01505

**Published:** 2025-04-14

**Authors:** Ali Maruf, Małgorzata Milewska, Katarzyna Dudzisz, Anna Lalik, Sebastian Student, Anna Salvati, Ilona Wandzik

**Affiliations:** † Department of Organic Chemistry, Bioorganic Chemistry and Biotechnology, Faculty of Chemistry, Silesian University of Technology, Krzywoustego 4, Gliwice, 44-100 Poland; ‡ Biotechnology Center, Silesian University of Technology, Krzywoustego 8, Gliwice 44-100, Poland; § Joint Doctoral School, Silesian University of Technology, Akademicka 2A, Gliwice 44-100, Poland; ∥ Department of Systems Biology and Engineering, Faculty of Automatic Control, Electronics and Computer Science, Silesian University of Technology, Akademicka 16, Gliwice 44-100, Poland; ⊥ Department of Nanomedicine & Drug Targeting, Groningen Research Institute of Pharmacy, University of Groningen, A. Deusinglaan 1, Groningen 9713AV, The Netherlands

## Abstract

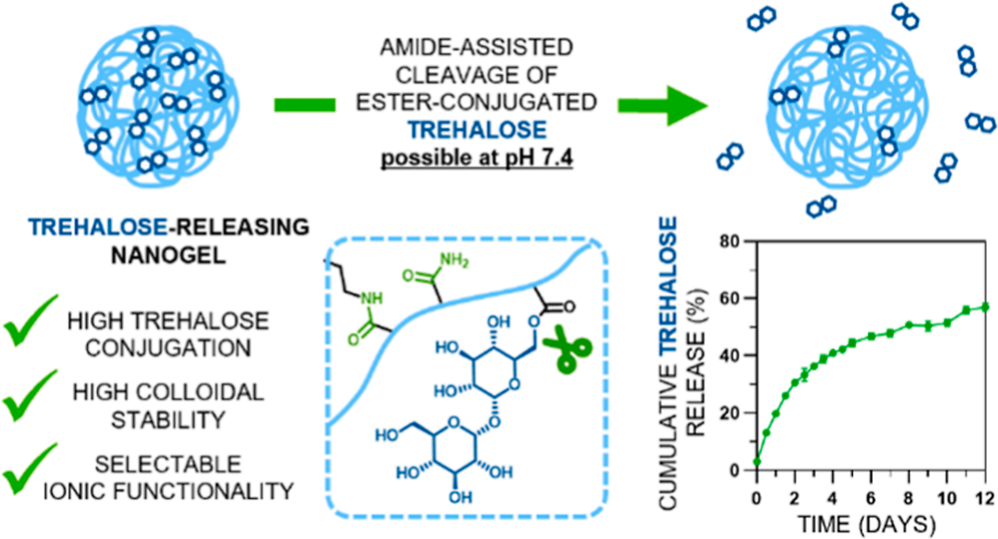

Trehalose has sparked considerable interest in a variety of pharmaceutical applications as well as in cryopreservation. Recently, there have been growing efforts in the development of trehalose delivery nanocarriers to address the issue of the poor bioavailability of trehalose. The majority of the strategies comprise physical entrapment of trehalose, since its covalent, yet biolabile, conjugation is challenging. Here, we present research on trehalose-releasing nanogels, in which covalent, yet biolabile, conjugation of trehalose was achieved through the co-incorporation of trehalose (meth)acrylate(s) together with hydrophilic primary/secondary acrylamides in one polymeric network. In this case, the primary and secondary amide groups participated in ester hydrolysis in the (meth)acrylate units, making the hydrolysis feasible under physiologically relevant conditions. A set of nanogels with precisely selected compositions were synthesized, characterized, and then studied to evaluate the influence of various structural and environmental factors on the release rate of trehalose. The study also provides insights into some other aspects that are important in view of potential biomedical applications, including specific interactions of nanogels through their terminal α-d-glucopyranosyl moieties from pendant trehalose, protein corona formation, and cellular uptake.

## Introduction

Over the past few years, there has been a growing interest in the use of trehalose, a naturally occurring disaccharide, in pharmaceutical applications. This interest is primarily due to trehalose’s ability to induce autophagy and provide neuroprotection by preventing protein aggregation.^1^ These findings have positioned trehalose as a promising therapeutic to target autophagy dysfunction and protein aggregation diseases.^2^ Unfortunately, trehalose has poor bioavailability due to its hydrophilic nature and susceptibility to enzymatic degradation.^3^

To address this problem, trehalose-containing nanocarriers in which trehalose is incorporated, either by physical entrapment or chemical conjugation, have gained popularity and emerged as an alternative option to free trehalose to improve its efficacy.^4^ This approach enhances trehalose’s effectiveness by protecting it from degradation and improving its delivery to target tissues. The physical entrapment of trehalose in a nanocarrier was shown to be effective for the stimulation of autophagy to treat atherosclerosis^5^ and for autophagy-enhanced cancer-cell ferroptosis.^6^ The physical encapsulation of trehalose into carrier systems is convenient, but the effectiveness of such a solution is moderate due to the characteristics of trehalose, e.g., the lack of charge, small size, and good water solubility. These features influence the poor retention of trehalose inside the carrier and, ultimately, the limited control over trehalose release. Alternatively, covalent binding of trehalose to the carrier system could effectively maintain the desired therapeutic concentration, resulting in an extended plasma half-life. In this strategy, trehalose needs to be chemically modified in a specific way that ensures successful conjugation with a carrier on the one hand and its subsequent sustained release on the other. The appropriate choice of biohydrolyzable trehalose conjugation could ensure control over the release from the carrier and consequently ensure its efficacy. Unfortunately, trehalose is a nonreducing disaccharide containing exclusively hydroxyl functional groups, two of which are primary hydroxyl groups. The lack of reducing properties and absence of other functional groups in trehalose significantly limits the possibilities of covalent and, at the same time, labile conjugations of trehalose with a potential carrier. To date, only a few examples of nanocarriers containing covalently bound and releasable trehalose have been reported. In the first report, a nanoassembly from trehalose-squalene conjugates linked via a biologically labile disulfide bond was demonstrated by the Seneci group for autophagy stimulation in vitro.^7^ Another strategy for trehalose-releasing nanosystems was developed in our group and concerned acrylamide-type nanogels with ester-linked trehalose.^8,9^ Two independent biological studies confirmed the autophagy stimulation effects of trehalose-containing nanogels. First, nanogels were capable of inducing autophagy in transgenic zebrafish and *Drosophila* larvae.^8^ Second, nanogels demonstrated the therapeutic effects of autophagy stimulation by promoting lipid efflux and plaque reduction in a mouse model of atherosclerosis.^9^

Among the numerous biological applications of trehalose, there is one particular use where trehalose delivery is necessary. Trehalose is known as a particularly attractive nonpermeable cryoprotective agent due to its role in protecting cells from various forms of damage.^10−12^ Optimal protection against stress and damage during cryopreservation necessitates the presence of trehalose in both the intracellular and extracellular compartments of cells.^13^ However, the challenge lies in the difficulty of transporting trehalose across the cell membrane, which limits its cryoprotective efficacy. Consequently, current research in this field is focused on developing delivery methods for trehalose into cells, and, again, trehalose carriers capable of facilitating its transport across the membrane are possible solutions. Three examples of trehalose carriers for the encapsulation of trehalose and intracellular transport for more effective cryopreservation are pH-responsive genipin-cross-linked Pluronic F127-chitosan nanoparticles,^14^ cold-responsive nanoparticles based on poly(*N*-isopropylacrylamide-*co*-butyl acrylate),^15^ and nanoparticles of chitosan–tripolyphosphate.^16^

Nanocarriers with various nanoscale architectures in which trehalose is physically encapsulated or chemically conjugated could be used to transport it into cells. Various colloidal polymeric systems, e.g., dendrimers, micelles, nanogels, nanoemulsions, and polymersomes, have been proposed as drug carriers. Among them, nanogel systems are gaining considerable attention due to their valuable properties, such as high loading capacity, softness, possible stimuli-responsive behavior, and generally good colloidal stability.^17^ Moreover, nanogels show significant promise in facilitating the transportation of drugs through the blood–brain barrier and blood–cerebrospinal fluid barrier and thus show potential for the delivery of drugs to the central nervous system.^18,19^ Nanogel conjugation with drugs and targeting ligands for brain targeting have been proposed. Examples include a nanogel system for insulin delivery, used as a protective agent in Alzheimer’s disease,^20^ and a cisplatin-loaded nanogel conjugated with a monoclonal antibody specific to brain transporters proposed as an antitumor treatment.^21^

Given the need for trehalose delivery, either for treating autophagy-related neurodegenerative disorders or to help trehalose to reach the intracellular space for more efficient cryopreservation, our attention was directed toward developing trehalose-releasing nanocarriers. Trehalose is conjugated in various nanogel compositions via a biohydrolyzable ester linkage, affording a nanoplatform for systemic pH-activated trehalose release. These studies complement our existing research on acrylamide-based (nano)hydrogels containing trehalose^8,9,22^ and allow previous findings to be systematized while also providing some new insights into the dependence of trehalose release on the nanogel composition. The main variables in the design are the content and structure of acrylamide-type units, the type of trehalose derivatives used for conjugation, and the ionic functionality. Trehalose-releasing nanogels with hydrolytically degradable croslinks were also developed. Furthermore, a nanogel releasing another disaccharide, sucrose, instead of trehalose, was developed as a potential counterpart for comparative studies. Following characterization of these nanogels, this study also investigated several new aspects that are important in the context of their potential biomedical applications. These included an investigation into the possibility of specific interactions of nanogels through the terminal α-d-glucopyranosyl moieties of the pendant trehalose, a study of their colloidal stability in serum-containing media, and protein corona formation, as well as deeper investigations into cellular uptake.

## Experimental Section

### General Methods

Ultrasonication was carried out on ice by using Sonics VCX 130 equipped with a 3 mm stepped microtip (Sonics & Materials, Inc., USA). All nanogels dispersions were prepared by ultrasonication at 40% amplitude for 30 s. Before the ultrasonication, nanogels were preswelled for 15 min.

NMR spectra were recorded in deuterated solvents (Deutero GmbH) on a Varian NMR instrument operating at 600 MHz. Chemical shifts are reported in ppm (δ) relative to the solvent residual signal or tetramethylsilane (CDCl_3_, DMSOd_6_) or 3-(trimethylsilyl)propionic-2,2,3,3-d_4_ acid sodium salt (D_2_O) as an internal reference. Deutered solvents were purchased from Deutero GmbH.

Trehalose or sucrose determinations were performed enzymatically based on standard curves by using a Trehalose Assay Kit, K-TREH or Sucrose/d-Fructose/d-Glucose Assay Kit, K-SUFRG (Megazyme International, Ireland), respectively, and following a microplate assay procedure, by using a Tecan Sunrise Microplate Reader.

Freeze drying was carried out under 0.035 mbar at −50 °C (ALPHA 1–2 LDplus, CHRIST).

Deionized water (DI water) was produced using a reverse osmosis system (σ < 2 μS/cm).

### Materials and Reagents for Synthesis and Characterization of Nanogels

6-*O*-Acryloyl-trehalose (TreA), 6,6′-di-*O*-acryloyl-trehalose (TreDA), 6′-*O*-acryloyl-sucrose (SucA), and 4-acrylamidobutanoic acid 3-sulfo-*N*-hydroxysuccinimide ester sodium salt (AMBA-sulfo-NHS) were synthesized following our previously described methods.^9,22,23^ 6-*O*-Methacryloyl-trehalose (TreMA) was synthesized following the same method as for TreA, except using methacryloyl chloride for acylation. Acrylamidobutanoic acid (AMBA) was synthesized according to the reported procedure.^24^ Acrylamide (AM, Acros Organics), *N*,*N′*-methylenebis(acrylamide) (MBAM, Acros Organics), 3-acrylamidopropyltrimethylammonium chloride (AMPTMAC, 75% w/w in H_2_O, Sigma-Aldrich), *N*-[3-(dimethylamino)propyl]-acrylamide (DMAP, TCI), 3-[(3-acrylamidopropyl)dimethylammonio]propane-1-sulfonate (DAPS, TCI), lithium phenyl (2,4,6-trimethylbenzoyl)phosphinate (LAP, Carbosynth), sulfo-Cy5-amine (Lumiprobe), 4-(2-hydroxyethyl)-1-piperazineethanesulfonic acid (HEPES; Acros Organics), Span 80 (Sigma-Aldrich), acetone (Chempur), cyclohexane (Chempur), DMSO (Extra Dry, AcroSeal, Acros Organics), triethylamine (TEA; Acros Organics), calcium chloride (CaCl_2_; POCH S.A.), disodium hydrogen phosphate heptahydrate (Na_2_HPO_4_·7H_2_O; Sigma-Aldrich), hydrochloric acid (HCl, 35–38%, POCH S.A.), manganese(II) chloride tetrahydrate (MnCl_2_·4H_2_O; Sigma-Aldrich), orthophosphoric acid (H_3_PO_4_, ≥85%, Merck), potassium chloride (KCl, Sigma-Aldrich), sodium chloride (NaCl; POCH S.A.), sodium dihydrogen phosphate monohydrate (NaH_2_PO_4_·H_2_O; Sigma-Aldrich), sodium hydroxide (NaOH; Sigma-Aldrich), and Concanavalin A (ConA) from Canavalia ensiformis (Jack bean) type-IV (Sigma-Aldrich) were used directly without any purification. Dialysis tubes (Spectra/Por 2 RC Dialysis Membrane, MWCO: 12–14 kDa) were purchased from Spectrum Laboratories, Inc.

### Synthesis of Nanogels

Nanogels were synthesized via an inverse miniemulsion free-radical polymerization according to our previously described method.^8^ Briefly, a water-in-oil (w/o) miniemulsion (1:10, v/v) was created from monomers and photoinitiator-containing PBS (pH 6.0, 1.0 mL) as the aqueous phase and Span 80-containing cyclohexane (0.6 g in 10.0 mL) as the organic phase. Monomers and cross-linker (amounts specified in Table S1) were placed in a 4 mL dark vial and dissolved in PBS (pH 6.0) to a total volume of 1 mL. Following the addition of the LAP initiator solution (2.3 mg in 51 μL), the aqueous phase was moved into a 20 mL clear vial containing the cold organic phase (4 °C), and the mixture was ultrasonicated in an ice bath for 5 min at 60% amplitude to create the miniemulsion. The walls of the reaction vial were then wrapped in aluminum foil and the vial was subjected to high-power light-emitting diodes (LEDs, 3 W, 395–405 nm) photoirradiation from the bottom for 0.5 h. The product was precipitated in 40 mL of acetone, centrifuged for 10 min at 14610*g*, washed twice with 40 mL of acetone, and air-dried overnight. The crude nanogel was purified by 24 h-long dialysis (MWCO 12–14 kDa) against water acidified with H_3_PO_4_ to pH 5.0 with multiple media changes and DI water as the last change. Finally, the nanogel dispersion was freeze-dried to yield a white fluffy powder and stored at 4 °C.

### Synthesis of Cy5-Labeled Nanogels

Fluorescently labeled nanogels were prepared by conjugating sulfo-Cy5-amine to active ester-bearing nanogels. Nanogels with active ester moieties were synthesized according to the procedure described above, except that the monomer composition was enriched with AMBA-sulfo-NHS monomer (amounts specified in Table S1), and the crude nanogel was not dialyzed. To carry out the conjugation, active ester-bearing nanogel (10.0 mg) was dispersed in anhydrous DMSO (250 μL) containing sulfo-Cy5-amine (0.25 mg, 0.00033 mmol) and triethylamine (0.068 mg, 0.00067 mmol), and the formed suspension was then left overnight with orbital shaking (1000 rpm, 25 °C). On the next day, the volume was increased to 1.0 mL with DMSO, and the crude nanogel was precipitated with 3.6 mL of acetone. The suspension was then centrifuged at 14610*g* (4 °C, 2 min) and washed six times with acetone (until the supernatant became colorless). The nanogel precipitate was then redispersed in 800 μL of DI water, ultrasonicated at 40% amplitude (30 s), and then dialyzed in dialysis capsules (QuixSep, MWCO 12–14 kDa) against water acidified with H_3_PO_4_ to pH 5.0 for 24 h with multiple media changes and DI water as the last change. Finally, the nanogel dispersion was freeze-dried to yield a blue fluffy powder and stored at 4 °C.

### Physicochemical Characterization of Nanogels

#### Hydrodynamic Diameter and ζ Potential Determination

The Z-average mean hydrodynamic diameter (d_H_) was determined by Dynamic Light Scattering (DLS) (Malvern, Zetasizer Nano 90S; 4 mV He–Ne ion laser, λ = 633 nm, scattering angle: 90°) for 1.0 mg/mL nanogel dispersion in DMEM at 37 °C. The samples were prepared from 10.0 mg/mL nanogel stock dispersion in DI water by diluting it with DMEM (without resonication). Before the measurement, samples were preincubated at 37 °C for 15 min.

The ζ potential of nanogels was measured by Electrophoretic Light Scattering (ELS) (Malvern, Zetasizer Nano ZC) for 1.0 mg/mL nanogel dispersion in 1 mM KCl.

#### Cryo-TEM Imaging

Cryo-TEM analysis was carried out using a Tecnai F20 X TWIN microscope (FEI Company, Hillsboro, Oregon, USA). Images were recorded with a Gatan Rio 16 CMOS 4k camera (Gatan Inc., Pleasanton, California, USA) and processed with Gatan Microscopy Suite (GMS) software (Gatan Inc., Pleasanton, California, USA). Specimens were prepared from nanogel dispersion in DI water (500 μg/mL) via the vitrification of aqueous solutions on oxygen plasma-activated grids with holey carbon film (Quantifoil R 2/2; Quantifoil Micro Tools GmbH, Groβlöbichau, Germany). Prior to use, the grids were activated for 15 s in oxygen plasma using a Femto plasma cleaner (Diener Electronic, Ebhausen, Germany). Cryo-TEM samples were prepared by applying a droplet (3 μL) of the nanogel dispersion to the grid, blotting with filter paper and immediately freezing in liquid ethane using a fully automated blotting device (Vitrobot Mark IV, Thermo Fisher Scientific, Waltham, Massachusetts, USA). Once prepared, the vitrified specimens were kept under liquid nitrogen until they were inserted into a cryo-TEM holder (Gatan 626, Gatan Inc., Pleasanton, USA) and analyzed in the TEM at −178 °C.

#### Determination of Trehalose* Content in Nanogels (CTre)

The content of trehalose in nanogels was determined enzymatically by using the Trehalose Assay Kit after pretreating nanogels with strong alkali, upon which ester bonds in acrylate units are hydrolyzed and all trehalose is liberated into solution and gets enzymatically detectable. Briefly, 40 μL of 1 M NaOH was added to 400 μL of nanogel dispersion (100 μg/mL) in PBS (pH 7.4), and the mixture was incubated at 70 °C for 1 h. After neutralization with 40 μL of 1 M HCl, the sample was subjected to enzymatic determination. CTre (% w/w) was calculated as the percentage of weight of trehalose in nanogel vs weight of nanogel. *Sucrose content was determined analogously by using the Sucrose/d-Fructose/d-Glucose Assay Kit.

#### Trehalose* Release Study by Enzymatic Determination

The nanogel stock dispersion in DI water (10.0 mg/mL) was diluted to a final concentration of 100 or 1000 μg/mL in PBS (pH 7.4, 6.5, or 8.0) containing a 1% v/v antibiotic antimycotic solution. Following the withdrawal of the first aliquot (800 μL), the nanogel dispersion was placed in an incubator at 37 °C with constant orbital shaking (332*g*). Aliquots (800 μL) were taken every 12 h for 5 days and then every 24 h from the 5th to 12th day and frozen at −20 °C. After all samples were collected, they were thawed and trehalose was determined enzymatically by using the Trehalose assay Kit.*Sucrose release was followed analogously by using the Sucrose/d-Fructose/d-Glucose Assay Kit.

Note: Only trehalose/sucrose which is liberated into solution is enzymatically detectable. Trehalose/sucrose which is covalently bound with nanogels cannot be hydrolyzed by trehalase enzyme and thus is not determined.

#### Trehalose Release Study by ^1^H NMR Spectroscopy

Nanogel lyophilizate (10 mg) was suspended in PBS (pH 7.4) in D_2_O containing a 1% v/v antibiotic antimycotic solution (1 mL) and sonicated to form a dispersion. Afterward, 700 μL of the nanogel dispersion was transferred to an NMR tube, and the ^1^H NMR spectrum was recorded (0 h). Afterward, the NMR tube was placed in an incubator at 37 °C under continuous orbital shaking (332*g*), and at prescribed time intervals (24, 72, and 168 h), the tube was withdrawn to acquire the ^1^H NMR spectrum. The presented spectra are normalized to the signal at ∼3.15 ppm corresponding to – CH_3_ protons from AMPTMAC units.

#### Determination of ζ Potential upon Trehalose Release

The nanogel stock dispersion in DI water (10.0 mg/mL) was diluted to 1.5 mg/mL in PBS (pH 7.4) containing a 1% v/v antibiotic antimycotic solution. Following the withdrawal of the first aliquot (800 μL), the nanogel dispersion was placed in an incubator at 37 °C with constant orbital shaking (332*g*). Aliquots (800 μL) were taken after 3, 6, 9, and 12 days of incubation and frozen at −20 °C. The samples with 100% of released trehalose were prepared from freshly dispersed nanogel (1.5 mg/mL in PBS pH 7.4, 1.0 mL), by adding 100 μL of 1 M NaOH, followed by incubation at 70 °C for 1 h, and neutralization with 100 μL of 1 M HCl. All samples (including thawed samples from trehalose release study) were then transferred to dialysis capsules (QuixSep, MWCO 12–14 kDa, 800 μL) and dialyzed against DI water acidified with H_3_PO_4_ to pH 5.0 for 24 h with multiple media changes and 1 mM KCl as the last change. Following the dilution of dialyzed samples to 1.0 mg/mL with 1 mM KCl, they were subjected to the ζ potential measurement.

#### Quantitative Precipitation of ConA

The ConA solutions were prepared by dissolving the lectin in 100 mM HEPES buffer (pH 7.2) containing 50 mM NaCl, 1 mM MnCl_2_, and 1 mM CaCl_2_ and passing it through a 0.20 μm syringe filter (CHROMAFIL H-PTFE Xtra). The solutions were prepared at 2.0 mg/mL to examine the nanogel concentration dependence and at 0.5, 1.0, 2.0, and 4.0 mg/mL to study ConA concentration dependence. Nanogels dispersions were prepared in the corresponding buffer at four concentrations (500, 250, 100, or 50 μM) considering the trehalose/sucrose concentration. Then, 500 μL of the nanogel dispersion was added to 500 μL of the ConA solution, gently mixed with a pipet, and incubated at 25 °C for 2 h. A control solution was prepared in the same way by replacing the nanogels dispersion with the corresponding buffer solution. After incubation, the white suspensions were centrifuged (12400*g*, 5 min, 25 °C), and 900 μL of the supernatant was collected and analyzed spectrophotometrically at 280 nm to determine the ConA concentration. The accurate ConA concentration was calculated based on A_1%, 1 cm_ = 13.7 at pH 7.2.^25^ The percentage of precipitated ConA was calculated from the difference between the ConA concentration in the control and in the sample. Each sample was analyzed in triplicate, and the reported values represent the mean value ± the standard deviation.

### Nanogels Interactions with Serum

#### Colloidal Stability in a Serum-Enriched Medium

Colloidal stability of nanogels in a serum-enriched medium (DMEM +10% FBS) was examined by DLS. The samples were prepared from 10.0 mg/mL nanogel stock dispersion in water by diluting it to 1.0 mg/mL with DMEM +10% FBS medium (without resonication). Before the measurement, samples were preincubated at 37 °C for 15 min. To assess one-day colloidal stability, the measurement was repeated after incubating the samples at 37 °C for 24 h (with orbital shaking, 332*g*).

#### Protein Corona Formation and Isolation of Corona-Coated Nanogels

Cy5-labeled cationic, anionic, and zwitterionic trehalose-releasing nanogels (NG5, NG6, and NG7) were incubated with full fetal bovine serum (FBS) (Gibco Thermo Fisher Scientific), at a nanogel concentration of 500 μg/mL, for 1.5 h under gentle shaking (250 rpm, 37 °C). The corona-coated nanogels were separated from the excess serum proteins by size exclusion chromatography (SEC) using a Sepharose CL-4B (Sigma-Aldrich) column (15 × 1.5 cm) prebalanced with PBS. To determine the elution profiles of protein absorption and nanogel fluorescence, fractions of 500 μL eluent were collected up to a total volume of 15 mL (30 fractions). Their absorbance (at 280 nm) and fluorescence (at *λ*_ex_/*λ*_em_ = 640/681 nm) were measured using a BioTek Synergy H1 multi-microplate reader. After evaluating the eluent profiles of protein absorbance and nanogel fluorescence, the isolated fractions of corona-coated nanogels were combined in a 2 mL Eppendorf tube and then centrifuged at 20500*g* for 1.5 h at 14 °C. The supernatant was discarded and then washed twice with PBS (10 min of centrifugation each wash). The protein and nanogel concentrations were calculated using a standard curve of BSA with different concentrations (0.047, 0.093, 0.188, 0.375, 0.75, 1.5, and 3 mg/mL, measured with the Bio-Rad DC Protein Assay kit) and Cy5-labeled nanogels with different concentrations (15.6, 31.3, 62.5, 125, 250, and 500 μg/mL), respectively (Figure S1). The protein corona was calculated as follows
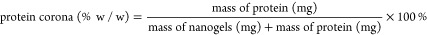


#### SDS-PAGE Gel Electrophoresis for Visualization of Protein Corona Components

In order to visualize protein corona for isolated corona-coated nanogels, the nanogels were mixed with loading buffer (containing 200 mM Tris–HCl, 400 mM DTT, 8% SDS, 0.4% bromophenol blue, and 40% glycerol) and heated for 5 min at 95 °C. Then, ∼60 μg of nanogels was loaded onto a freshly prepared 10% polyacrylamide gel together with samples of FBS and FBS fractions (∼30 μg, as control) and run for 1 h at 120 V at room temperature. The gels were stained by using a solution containing 0.1% Coomassie blue R-250 in a water–methanol–glacial acetic acid (5:4:1) mixture with gentle agitation, followed by destaining in hot ultrapure water. Images were captured using a ChemiDoc XRS (Bio-Rad).

### Uptake Behavior and Mechanisms of Nanogels in HeLa Cells

#### Cell Culture

HeLa CCL-2 cells were purchased from ATCC (Manassas, VA, USA). Complete cell culture medium (cMEM) consisting of MEM (Gibco Thermo Fisher Scientific, Landsmeer, the Netherlands) or DMEM/F12 (PAN Biotech) supplemented with 10% v/v Fetal Bovine Serum (FBS, Gibco Thermo Fisher Scientific or FBS, EURx) was used to cultivate the cells. The cell culture was maintained at 37 °C with 5% CO_2_. After being defrosted, the cells were cultured for a maximum of 20 passages and were regularly checked to rule out mycoplasma infection.

#### Cell Uptake Kinetics

The uptake kinetics of cationic, anionic, and zwitterionic trehalose-releasing nanogels were assessed in HeLa cells. Briefly, HeLa cells were seeded at a concentration of 30000 cells/well in a 24-well plate (Greiner Bio-One BV, A. Alphen on den Rijn, the Netherlands). After 24 h of seeding, HeLa cells were incubated with Cy5-labeled cationic, anionic, and zwitterionic trehalose-releasing nanogels (Cy5-NG5, Cy5-NG6, and Cy5-NG7:1 μg/mL) for 29 h. At the predetermined time points (30 min, 2, 5, 7, 22, 25, and 29 h), cells were collected and prepared for flow cytometry. Controls (without nanogel treatment) were included in each time point. The samples were measured in fresh form (without fixation).

#### Study of the Mechanism of Uptake with Different Endocytosis Inhibitors

The mechanisms of uptake of cationic, anionic, and zwitterionic trehalose-releasing nanogels were characterized using inhibitors of endocytosis and previously optimized protocols to avoid toxicity. HeLa cells were seeded at a concentration of 50000 cells/well in a 24-well plate (Greiner Bio-One BV, A. Alphen on den Rijn, the Netherlands). After 24 h of seeding, HeLa cells were preincubated with various inhibitors. HeLa cells were initially preincubated with glucose-free media containing 50 mM of 2-deoxy-d-glucose (2-DG, a nonmetabolizable glucose analogue) and 0.02% v/v of NaN_3_ for 1 h in order to examine if the nanogels penetrate the cells via an energy-dependent route. HeLa cells were also preincubated with different endocytosis inhibitors: chlorpromazine hydrochloride (CP) (10 μg/mL in cMEM for 20 min), 5-(*N*-ethyl-*N*-isopropyl)amiloride (EIPA) (50 μM in cMEM for 20 min), and hydroxy-dynasore (Dyn) (2.5 μg/mL in cMEM for 30 min). All inhibitors were obtained from Sigma-Aldrich, St. Louis, USA. Afterward, cells were exposed to Cy5-labeled cationic, anionic, and zwitterionic trehalose-releasing nanogels (NG5: 0.1 and 2 μg/mL, NG6 and NG7: 2 μg/mL) dispersed in cMEM or in the presence of the inhibitors, for another 5 h. Controls (without nanogel treatment) were included in each experiment. An additional experiment was conducted in glucose-free media and cMEM to check the uptake behavior of nanogels compared to silica nanoparticles (SiNPs, size: ∼50 nm, as control), for 5 h uptake. Finally, cells were collected and prepared for flow cytometry.

#### Flow Cytometry Analysis

Flow cytometry was used to measure the fluorescence intensity of HeLa cells incubated with the Cy5-labeled nanogels. To get rid of any possible nanoparticles that were stuck to the cell membrane, cells were rinsed twice, once with cMEM and once with PBS. The cells were harvested using 0.05% trypsin–EDTA for 5 min at 37 °C, collected, centrifuged for 5 min at 400*g*, and then fixed with 250 μL of paraformaldehyde (PFA, 4%) for 20 min. Then, the cells were washed with 500 μL of PBS, centrifuged for 5 min at 400*g*, resuspended in 100 μL of PBS, and finally stored at 4 °C prior to the measurement. Cell fluorescence was recorded using a BD FACS array (BD Biosciences, Erembodegem, Belgium) using 638 and 561 nm lasers (Beckman Coulter, Woerden, the Netherlands) with detection on an APC-A channel (660/20 nm BP) for Cy5-labeled nanogels and a PE-A channel (585/42 nm BP) for DiI-labeled SiNPs, respectively. Data were analyzed using FlowJo data analysis software (FlowJo, LLC). Cell debris and cell doublets were excluded by setting gates in the forward and side scattering double scatter plots. A total of at least 15000 cells were acquired per sample and each sample was performed in duplicate. Then the average and standard deviation of the median cell fluorescence intensity were calculated. Experiments were repeated at least 3 times to confirm reproducibility. The results are the average and standard error of the average results obtained in 3 independent experiments (unless specified).

#### In Vitro Cell Uptake Study by Confocal Microscopy

HeLa cells were seeded at the density of 45000 cells/well in a μ-Slide 8 Well Glass Bottom chambered coverslip (ibidi, USA) in 200 μL of DMEM/F12 (PAN Biotech) supplemented with 10% FBS (EURx) and incubated at 37 °C for 24 h. Then, the medium was replaced with fresh DMEM/F12 (PAN Biotech) + 10% FBS (EURx) containing Cy5-labeled nanogels (100 or 10 μg/mL) and cultured for 5 h. Afterward, cell nuclei were stained with Hoechst 33342 (Thermo Fisher Scientific), and cell membranes were stained with MemBrite Fix 488/515 (Biotium) according to the manufacturer’s protocol. The distribution of nanogels in HeLa cells was examined by confocal laser scanning microscopy (CLSM, Olympus FluoView FV1000, ZEISS, Dublin, CA, USA).

#### Cytotoxicity by MTT Assay

HeLa cells were seeded at a concentration of 30000 cells/well in a 24-well plate (Greiner Bio-One BV, A. Alphen on den Rijn, the Netherlands). After 24 h of seeding, HeLa cells were treated with nanogels NG5, NG6, and NG7 at different concentrations (10, 100, 500, and 1000 μg/mL in cMEM) and cocultured for 24 h (37 °C). HeLa cells without treatment were used as control, while 10% H_2_O and 10% DMSO were used as the control vesicle and negative control, respectively, to ensure that the effects observed in the experiment are due to the nanogel treatments and not due to any other variables. Standard MTT assay was conducted to assess the cytotoxicity profile of nanogels. Briefly, the old media were removed and the cells were washed with 500 μL of cMEM. Then, the cells were incubated with 250 μL of MTT solution (concentration: 0.5 mg/mL in cMEM) for 30 min in the incubator (37 °C). After that, the media were removed and DMSO (250 μL) was given to each well and the plates were shaken for 15 min at room temperature. Then, 200 μL of solution from each well was transferred to a 96-well plate and the absorbance was measured with the BioTek Synergy H1 multi-microplate reader at 550 nm. The cell viability was calculated by normalizing the absorbance of each treatment to the control (without treatment) and presented as the percentage of living cells.

## Results and Discussion

### Design, Synthesis, and General Characteristics of Nanogels

**Figure 1:**
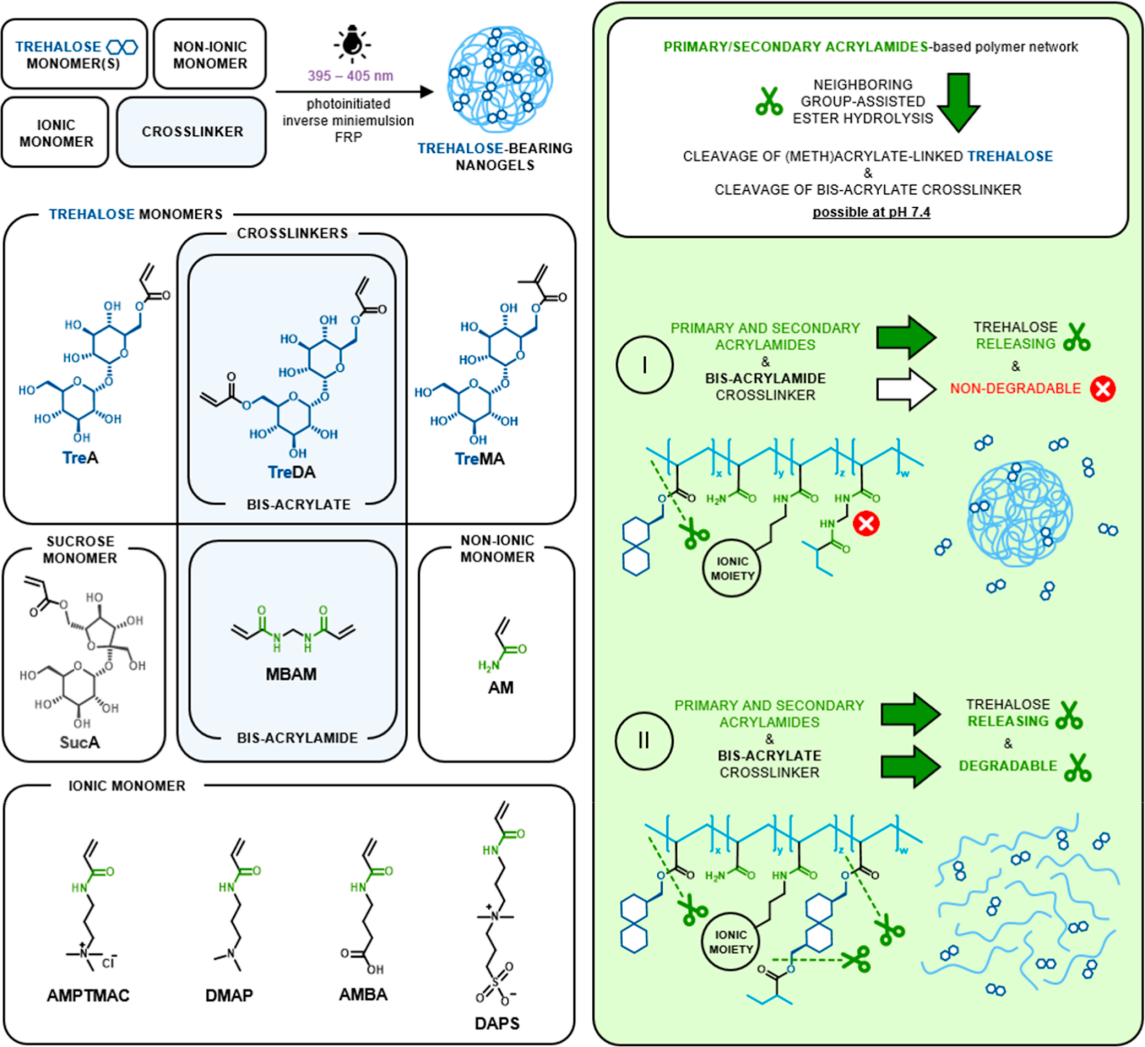
Monomers for the synthesis of trehalose-releasing nanogels (left). Basis of trehalose release from nanogels at systemic pH and chemical structure of the polymeric network of (I) trehalose-releasing/nondegradable nanogels and (II) trehalose-releasing/degradable nanogels.

The current approach for trehalose-releasing nanocarriers is based on the cleavage of ester-linked trehalose. Generally, the ability of the ester bond to form readily biocleavable drug-polymer conjugates is limited because without specific circumstances (enzyme assistance or specific structural features), the hydrolysis rates of esters at physiologically relevant conditions are generally very slow.^26^ The susceptibility of esters to hydrolysis can be strongly influenced by neighboring-group participation, which is a phenomenon involving direct interaction of an intramolecular substituent with the reaction center, leading to considerable enhancement in the reaction rate.^27^ Recently, we have found that neighboring-group participation in ester hydrolysis takes place in polymeric networks of hydrogels fabricated from acrylamides and acrylates.^28^ Specifically, the presence of primary and secondary acrylamide-type units significantly accelerates the hydrolysis of ester moieties in acrylate units, indicating the neighboring amide group participation in ester hydrolysis. Utilizing this effect and copolymerizing trehalose 6-*O*-acrylate (TreA) with selected acrylamides, it was possible to develop bulk^22^ and nanosized^8,9^ hydrogels, which can sustainably release trehalose at pH 7.4. In the current study, the research on trehalose-releasing nanogels is continued on nanogels of 11 different compositions (Table 1), which were synthesized from various trehalose (meth)acrylates and selected acrylamides (Figure 1, left). Depending on the cross-linker selection for synthesis, MBAM or TreDA, trehalose-releasing nanogels can be classified as (I) trehalose-releasing but nondegradable or (II) trehalose-releasing and degradable, respectively (Figure 1, right). Additionally, one sucrose-releasing nanogel was also synthesized.

**Table 1:** Monomer Feed Composition and Physicochemical Characteristics of Trehalose-Releasing Nanogels

nanogel	trehalose monomer (mmol)	nonionic monomer (mmol)	ionic monomer (mmol)	cross-linker (mmol)	CTre (% w/w)	*d*_H_ (PDI) in DMEM (nm)	ζ potential (mV)	colloidal stability in serum-containing DMEM^b^
**NG1**	TreA (0.204)		AMPTMAC (+) (0.605)	MBAM (0.130)	27.6	163 (0.21)	+38.0	A
**NG2**	TreA (0.341)		AMPTMAC (+) (0.341)	MBAM (0.130)	46.9	126 (0.18)	+41.5	A
**NG3**	TreA (0.412)		AMPTMAC (+) (0.206)	MBAM (0.130)	57.2	81 (0.19)	+25.5	S
**NG4**	TreA (0.341)	AM (0.497)	AMPTMAC (+) (0.171)	MBAM (0.130)	46.6	213 (0.26)	+32.9	A
**NG5**	TreA (0.385)	AM (0.497)	AMPTMAC (+) (0.085)	MBAM (0.130)	53.3	115 (0.21)	+30.2	S
**NG6**	TreA (0.385)	AM (0.497)	AMBA (−) (0.085)	MBAM (0.130)	57.6	57 (0.24)	–17.6	S
**NG7**	TreA (0.385)	AM (0.497)	DAPS (±) (0.085)	MBAM (0.130)	53.7	61 (0.22)	–9.6	S
**NG8**	TreA (0.385)	AM (0.497)	DMAP (+) (0.085)	MBAM (0.130)	47.3	125 (0.19)	+36.8	S
**NG9**	TreMA (0.385)	AM (0.497)	AMPTMAC (+) (0.085)	MBAM (0.130)	38.8	169 (0.30)	+29.1	S
**NG10**		AM (0.497)	AMPTMAC (+) (0.085)	TreDA (0.385)	54.0	A	+36.1	A
**NG11**	TreA (0.255)	AM (0.497)	AMPTMAC (+) (0.085)	TreDA (0.130)	62.9	67 (0.29)	+32.6	S
**NG12**	SucA^a^ (0.385)	AM (0.497)	AMPTMAC (+) (0.085)	MBAM (0.130)	46.4^a^	92 (0.20)	+34.0	S

a Refers to sucrose instead of trehalose.

b Refers to DMEM + 10% FBS; S–stable (lack of aggregation confirmed by DLS); A–rapidly aggregating.

The composition of trehalose-containing nanogels was carefully selected to study various effects. The **NG1–5** nanogels were synthesized by varying TreA, AMPTMAC (cationic and secondary acrylamide-type monomer, Figure 1), and AM (primary acrylamide-type monomer, Figure 1), using a constant amount of MBAM as a cross-linker. The aim was to provide insight into the dependence of trehalose release on acrylamide-type monomers—their molar content and the order of the amide group. From among them, **NG5** was selected as an optimal composition, which was further modified to study other effects. In **NG9**, TreA was replaced by its methacrylate analogue, TreMA, to assess whether trehalose release could be controlled by the structure of the acyl moiety through which the trehalose was incorporated within a nanogel network. The compositions of **NG6**, **NG7**, and **NG8** were modified to incorporate monomers with ionic/ionizable moieties, AMBA, DAPS, and DMAP (Figure 1), respectively, instead of the AMPTMAC used in **NG5**. In all, they enabled the effects of various ionic functionalities to be evaluated, including quaternary ammonium, tertiary ammonium, zwitterionic, and carboxylate. The **NG5**, **NG10**, and **NG11** nanogels differed in the way that they incorporated trehalose into the polymer network, whether as a mono- or diester or a combination of both, and hence in the number of its attachment points. **NG10** was synthesized with an equimolar amount of trehalose as in **NG5** but using trehalose diacrylate (TreDA) instead of its monoacrylate, while **NG11** was synthesized using a mixture composed of ∼70% of monoacrylate and ∼30% of diacrylate. Moreover, in **NG10** and **NG11**, TreDA acted simultaneously as a cross-linker, which fully replaced MBAM. This introduced labile ester moieties at the cross-linking points, theoretically making the nanogels hydrolytically degradable (Figure 1 right, (II)). Finally, the last nanogel, **NG12**, was designed to release other disaccharide instead of trehalose for comparative studies. Hence, in the composition of **NG12**, trehalose monomer was replaced by its sucrose-based analogue, sucrose acrylate (SucA). The latter monomer shares significant structural commonalities with the trehalose monomer, TreA, which makes them perfect analogues. Both TreA and SucA have exactly the same MW and contain the same number of –OH groups. They are both acryloylated on the primary –OH group, are nonreducing, and can introduce terminal α-d-glucopyranosyl moieties into the polymer structure.

**Figure 2:**
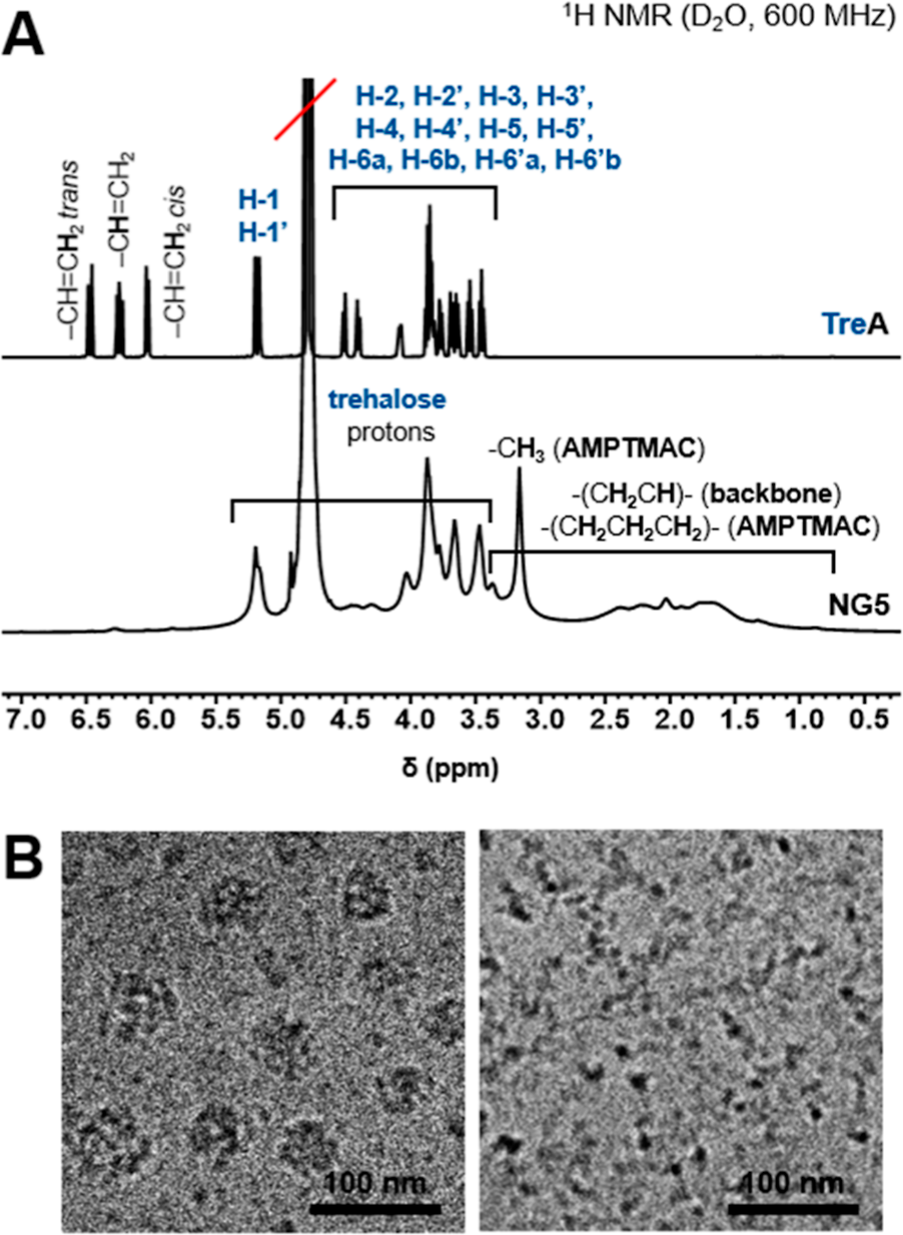
(A) ^1^H NMR spectra of TreA (top) and **NG5** (bottom) confirming covalent incorporation of trehalose into the nanogel (D_2_O, 600 MHz). (B) Cryo-TEM micrographs of **NG11** dispersion in PBS pH 7.4 before (left) and after 6 days (right) of incubation at 37 °C.

The nanogel synthesis was accomplished by a facile procedure involving photoinitiated free-radical polymerization (FRP) in a water/oil (w/o) miniemulsion with yields in the range from 61 to 85%. The successful covalent incorporation of trehalose and the nanogels’ purity were confirmed by ^1^H NMR spectroscopy, as shown by the example of **NG5** (Figure 2A). The presence of broad signals typical for polymers in the range of 3.3–4.6 and 5.0–5.5 ppm, which were well-correlated with the proton signals of the trehalose monomer (TreA), and the lack of signals from protons of the acrylate group in the range of 6.0–6.5 ppm both proved the successful incorporation of TreA into the nanogel network. Signals originating from the protons of other key structural fragments, e.g., methyl (3.0–3.3 ppm) from AMPTMAC, as well as the polymer backbone derived from acrylates and acrylamides (1.0–3.0 ppm), could also be easily identified.

Cryo-TEM imaging proved that the nanogels had a close to spherical shape (Figure 2B, left). The general physicochemical characteristics of the trehalose-containing nanogels, including the total content of incorporated trehalose (CTre), the d_H_ with PDI in DMEM, the ζ potential, and colloidal stability, are included in Table 1. CTre was generally well-correlated with trehalose monomer feeding, and for most nanogels, it exceeded 50% w/w. All the nanogels were well-dispersible and formed stable dispersions in DMEM, except for **NG10**. The poor colloidal stability of **NG10** is most likely due to its highly cross-linked structure, resulting from a high content of TreDa. The Z-average *d*_H_ for most nanogels determined in this medium was lower than 170 nm (PDI 0.18–0.30) but with no clear trends or correlations with other properties. Prominent differences in colloidal stability became apparent in serum-containing DMEM (DMEM + 10% FBS). Under these conditions, **NG1**, **NG2**, and **NG4** readily aggregated, forming sedimenting precipitates that were clearly visible to the naked eye. In turn, the rest of the nanogels (except for **NG10**) exhibited good dispersibility and colloidal stability in the presence of serum, with the absence of aggregation confirmed by DLS. Examples of DLS distributions of nanogels which are stable in DMEM + 10% FBS in comparison to DLS distributions in serum-free DMEM are presented in Figure 6 for **NG5**, **NG6**, and **NG7**. The ζ potential of the nanogels was clearly dependent on the type of ionic functionality and reached −17.6 mV for **NG6** (which had anionic carboxylic acid moieties), +36.8 mV for cationic nanogels containing tertiary amine groups (**NG8**), and ranged from +22.6 to +41.5 mV for cationic nanogels with quaternary ammonium cations (**NG1–5** and **NG9–12**). The slightly negative ζ potential (−9.6 mV) of **NG7,** which had zwitterionic units, resulted from the presence of carboxylate anions from the residual photoinitiator moieties.

### Trehalose Release

**Figure 3:**
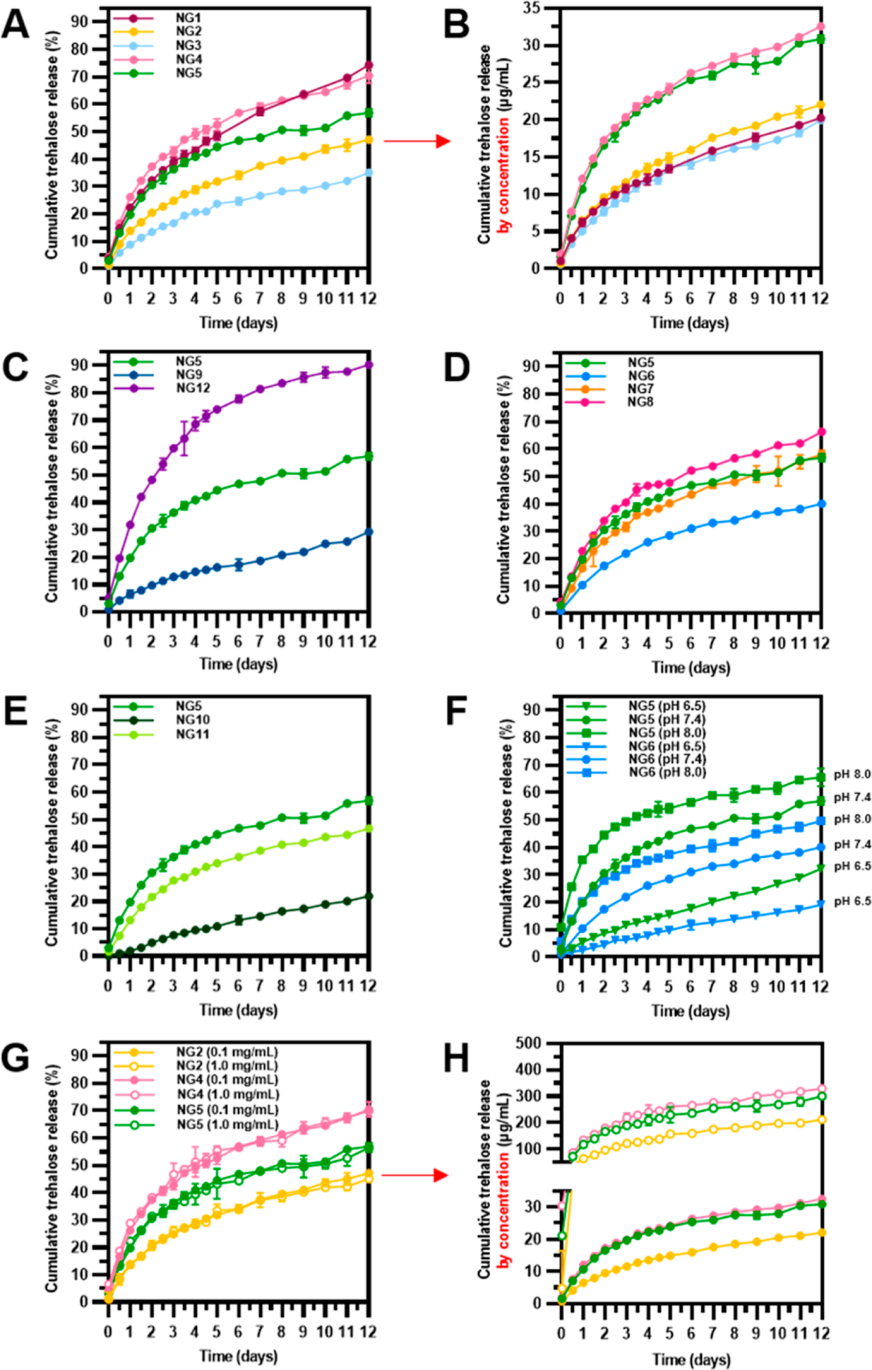
Trehalose release from trehalose-releasing nanogels. (A–E) Release profiles for all nanogels at systemic pH (PBS, pH 7.4, 37 °C; NG concentration: 100 μg/mL). (F) Release profiles for cationic nanogel **NG5** and anionic nanogel **NG6** at three biologically relevant pH: 6.5; 7.4; and 8.0 (PBS, 37 °C; NG concentration: 100 μg/mL). (G,H) Release profiles for selected nanogels at different NG concentrations (100 vs 1000 μg/mL_,_ PBS, pH 7.4, 37 °C; NG concentrations: 100 μg/mL). Data are presented as mean ± SD (*n* = 3).

The quantitative trehalose release was followed by enzymatic determination of trehalose, and the release profiles are presented in Figure 3. Apart from the percentage release, some concentration data are also presented. These two types of analysis provided deeper insight, as they highlighted two different aspects of trehalose release. While the percentage release allowed comparison of the release rates, the release presented in terms of concentration gave direct information about the absolute amount of released trehalose. Trehalose release profiles of first five nanogels (**NG1–5**), presented in Figure 3A,B, show clearly that both the content and the structure of the acrylamide-type comonomer significantly influenced its impact on the hydrolysis rate of ester moieties in the acrylate units and thus on trehalose release. In **NG1**, **NG2**, and **NG3,** the content of trehalose acrylate units increased, while that of the acrylamide-type unit AMPTMAC decreased, which translated to a considerable slowing down of the trehalose release rate (Figure 3A). Consequently, although the content of conjugated trehalose in **NG3** was twice as much as in **NG1** (∼57% vs ∼28%, respectively), the amount of released trehalose over time was almost the same (Figure 3B). This led to the conclusion that the lower the percentage of acrylamide-type monomer units in the polymeric network, the slower the ester-bonded trehalose was cleaved. Another important issue in the acceleration of ester hydrolysis by acrylamide-type units was that the effect of the primary amide moiety was more prominent than that of the secondary amide group. This relationship was clearly observed in the release profiles of **NG2** and **NG4**, which contained similar amounts of trehalose acrylate, but in **NG4,** half of the AMPTMAC was replaced by AM. The presence of AM significantly sped up the release rate, e.g., after 1 week, ∼25% more trehalose was released from **NG4** than from **NG2**. Unfortunately, although **NG4** was the best performing nanogel in terms of the amount of trehalose being released among all the discussed nanogels, it had poor colloidal stability in serum-enriched biological media, where it aggregated immediately. Excellent improvement in colloidal stability was achieved by increasing the content of trehalose acrylate in place of AMPTMAC in **NG5**. And despite the reduction in the percentage of acrylamide-type monomer units, causing a decrease in the release rate, the increased trehalose content ensured that the amount of trehalose being released from **NG5** was fairly similar to that being released from **NG4**. Thus, based on the trehalose release profiles and taking into account colloidal stability, **NG5** was selected as the optimal composition, which was then further modified to study other effects.

The structure of the acyl moiety through which trehalose was incorporated within the nanogel network also had a considerable impact on trehalose release. The percentage of trehalose released was ∼2–3 times lower from **NG9** containing trehalose methacrylate units in comparison to **NG5** with trehalose acrylate units (Figure 3C). Specifically, the percentage released from methacrylate- vs acrylate-containing nanogels reached ∼6% vs ∼20% after 1 day, ∼15% vs ∼45% after 6 days, and ∼25% vs ∼55% after 12 days, respectively. The observed differences are in agreement with the literature data, which indicate a higher resistance of methacrylate-based polymers to alkaline hydrolysis compared to acrylate-based polymers.^29,30^

Figure 3C also shows the release profile for **NG12,** containing sucrose in place of the trehalose incorporated in **NG5**. The release of sucrose from **NG12** was significantly faster than that of trehalose from **NG5**, reaching ca. 90% and 60% after 12 days, respectively. This result indicated that the 6′-*O*-ester of sucrose (involving the primary OH 6′–OH group from the fructofuranosyl moiety) was more prone to hydrolysis than the 6-*O*-ester of trehalose (involving the primary 6-OH group from the glucopyranosyl moiety). Unfortunately, although there are structural commonalities between sucrose-releasing nanogels and trehalose-releasing nanogels, the significant difference in the release rates rather excludes sucrose-releasing nanogels from serving as controls for trehalose-releasing nanogels in biological studies, particularly since the change in the nanogels’ charge following the release and formation of carboxylate moieties would be different, and thus, the nanogels’ characteristics would change differently.

The effect of various ionic functionalities on trehalose release is presented in Figure 3D. The release was the fastest from **NG8**, which had a cationic tertiary amino group, then slightly slower from **NG5** containing a quaternary ammonium group and from **NG7** containing a zwitterionic moiety, while it was the slowest from **NG6**, which had anionic carboxylate functionality, reaching ca. 65%, 55%, 55%, and 40% after 12 days, respectively. The observed differences could have potentially resulted from differences in local pH or ion mobility inside the nanogel network. A faster rate of ester hydrolysis in hydrogel networks containing positively charged moieties compared to those with negatively charged groups has been previously observed in the literature.^31^ Jo et al. demonstrated that hydrogel degradation proceeding through an ester moiety cleavage can be modulated by neighboring amino acids, with a ca. 12-fold faster hydrolysis in case of a network containing positively charged arginine units than that with negatively charged aspartic acid units. The most surprising of our results was the lack of substantial differences in the release profiles from **NG5** with quaternary ammonium moieties and **NG7** with zwitterionic functionalities. The determining factor here may be that both of them contain a quaternary ammonium cation, localized at the same distance from the polymer backbone.

**Figure 4:**
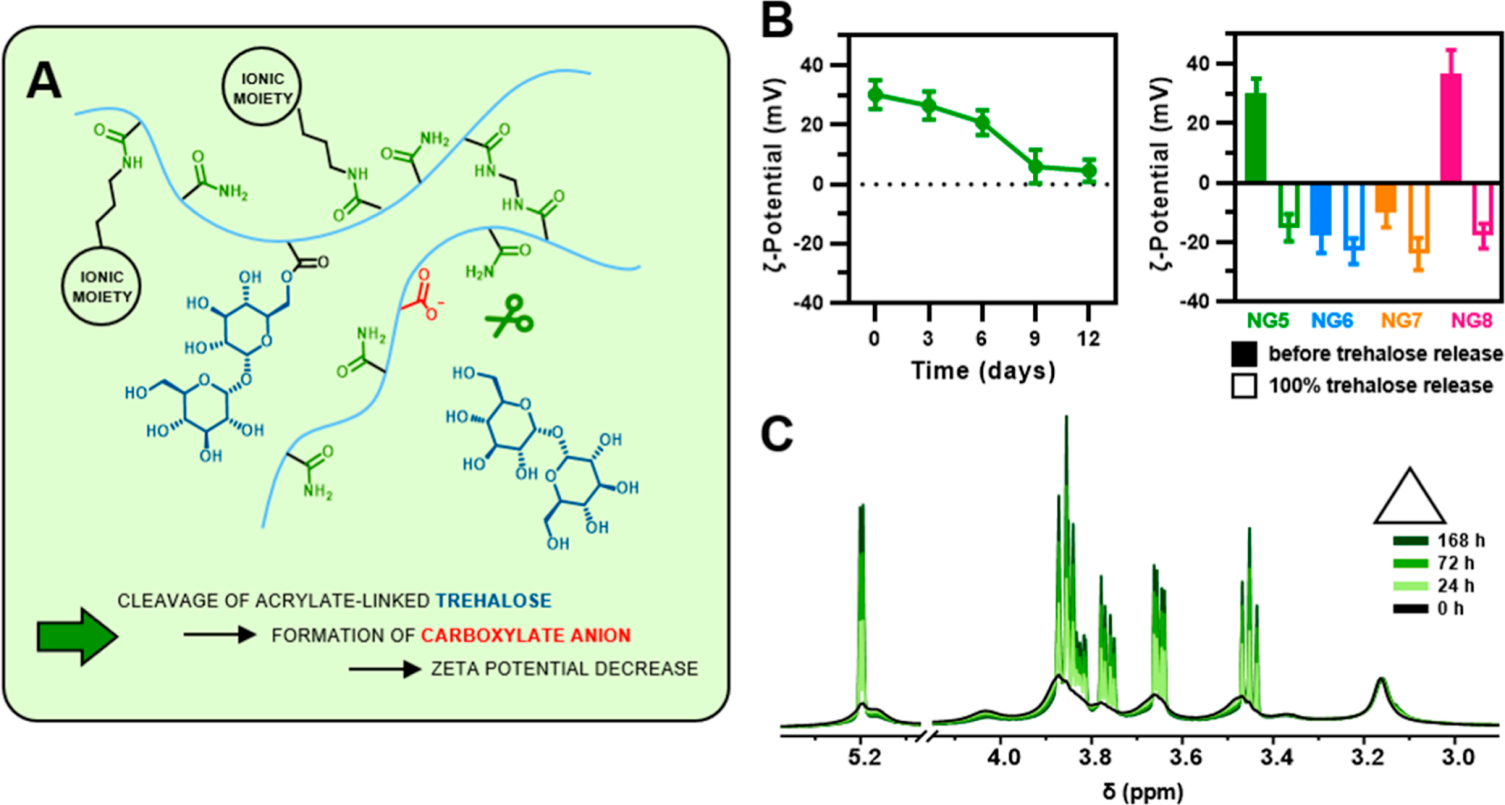
(A) Scheme of trehalose release from the primary/secondary acrylamides-based polymer network via the hydrolytic cleavage of the ester bond. (B) Change in ζ potential during trehalose release from **NG5** in PBS, pH 7.4, 37 °C (left). ζ potential of nanogels with various ionic functionalities before (filled) and after (hollow) complete trehalose release (right). (C) Section of ^1^H NMR spectra of **NG5** before and during the incubation in PBS (in D_2_O) at pH 7.4, 37 °C, showing sustained trehalose release.

The study on trehalose release from nanogels with various ionic functionalities was extended by an experiment involving measurement of the ζ potential upon trehalose release and also after full cleavage of trehalose. The formation of carboxylate ions following ester bond hydrolysis introduced a negative charge to the nanogel network (Figure 4A), which was expected to cause a decrease in ζ potential. Indeed, as shown on the example of **NG5** containing quaternary ammonium groups and characterized by an initial ζ potential of +30 mV, this value gradually decreased and reached +4 mV on the twelfth day of release (Figure 4B, left). Furthermore, the full cleavage of trehalose caused charge reversal and changed the initial ζ potential from +30 mV to −15 mV (Figure 4B, right). In the case of other functionalities, the ζ potential after the full cleavage of trehalose changed as follows: from +37 mV to −18 mV for **NG8** with cationic tertiary amino groups, from +10 mV to −24 mV for **NG7** with zwitterionic moieties, and from −18 mV to −23 mV for **NG6** with anionic carboxylate functionality (Figure 4B, right). The change in ζ potential was, however, more pronounced for nanogels with cationic groups than for those with zwitterionic or anionic groups, indicating a different degree of protonation of the formed carboxyl groups. In nanogels with cationic functionality, carboxylate anions can act as counterions for quaternary or tertiary ammonium cations, which keeps the carboxyl groups deprotonated. Nanogels with anionic functionality lack such cations, and it is likely that more of the carboxyl groups are protonated, so they do not contribute to the ζ potential.

Figure 3E presents the release profiles from nanogels where trehalose was incorporated as a mono- and/or diester. As expected, the greater the number of ester bonds to be cleaved, the slower was the release. Hence, the release was the fastest for **NG5**, containing only trehalose monoester, slower for **NG11,** which was synthesized using 70% of TreA and 30% of TreDA, and the slowest for **NG10**, where all the monoester was replaced by diester. The most important result here is that trehalose was released from **NG10**. This result confirmed that trehalose diacrylate can act as a hydrolytically labile cross-linker because trehalose is only detectable enzymatically once both ester bonds are cleaved and trehalose is released into solution. Cryo-TEM imaging further confirmed the possibility of providing the degradability of acrylamide-based nanogel through trehalose diacrylate cross-linking. After a six-day-long incubation of **NG11** dispersion in PBS (pH 7.4) at 37 °C, nanogel disintegration was clearly observable (Figure 2B).

The final studies on trehalose release were focused on assessing the effects of nanogel concentration and solution pH. Figures 3G and 3H present release profiles (by percentage and by concentration, respectively) for three selected nanogels, **NG2**, **NG4**, and **NG5**, at two concentrations differing by 1 order of magnitude: 0.1 and 1.0 mg/mL. For all three nanogels, the amount of released trehalose was proportional to the nanogel concentration and differed ∼10 times between these two concentrations, but the release rate was not influenced by concentration. These results indicate that the hydrolysis rate is determined by the “local” concentration of ester moieties within one nanogel particle rather than their “global” concentration in the dispersion.

The influence of pH was studied for two oppositely charged nanogels: **NG5** with quaternary ammonium cations and **NG6** bearing carboxylic acid groups. The release of trehalose was compared for three different pH values, 6.5, 7.4, and 8.0, which are considered as biologically relevant. Both nanogels exhibited clear pH-dependent trehalose release rates, which were faster the higher the pH (Figure 3F). Furthermore, regardless of the pH, the release was always faster from the cationic nanogel than from the anionic nanogel.

Finally, it is worth noting that none of the release profiles followed pseudo-first-order kinetics, which is frequently assumed for hydrolysis-driven release. This result indicates that trehalose liberation is more complex, and hydrolysis is influenced by a number of factors, one may be the changing composition of ionic moieties. Ionic moieties were shown to influence the release rate, thus increasing content of carboxylate anions in the nanogel network along with trehalose release probably affected further release.

The sustained release of trehalose from the nanogels at pH 7.4 (37 °C) was also clearly reflected in the ^1^H NMR spectra, as shown by the example of **NG5** (Figure 4C). The intensity of the sharp peaks corresponding to the protons of free trehalose increased with increasing incubation time, while the broad peaks originating from protons of trehalose still bound to the nanogel network decreased.

### Specific Interactions of Nanogels through α-d-Glucopyranosyl Residues

**Figure 5:**
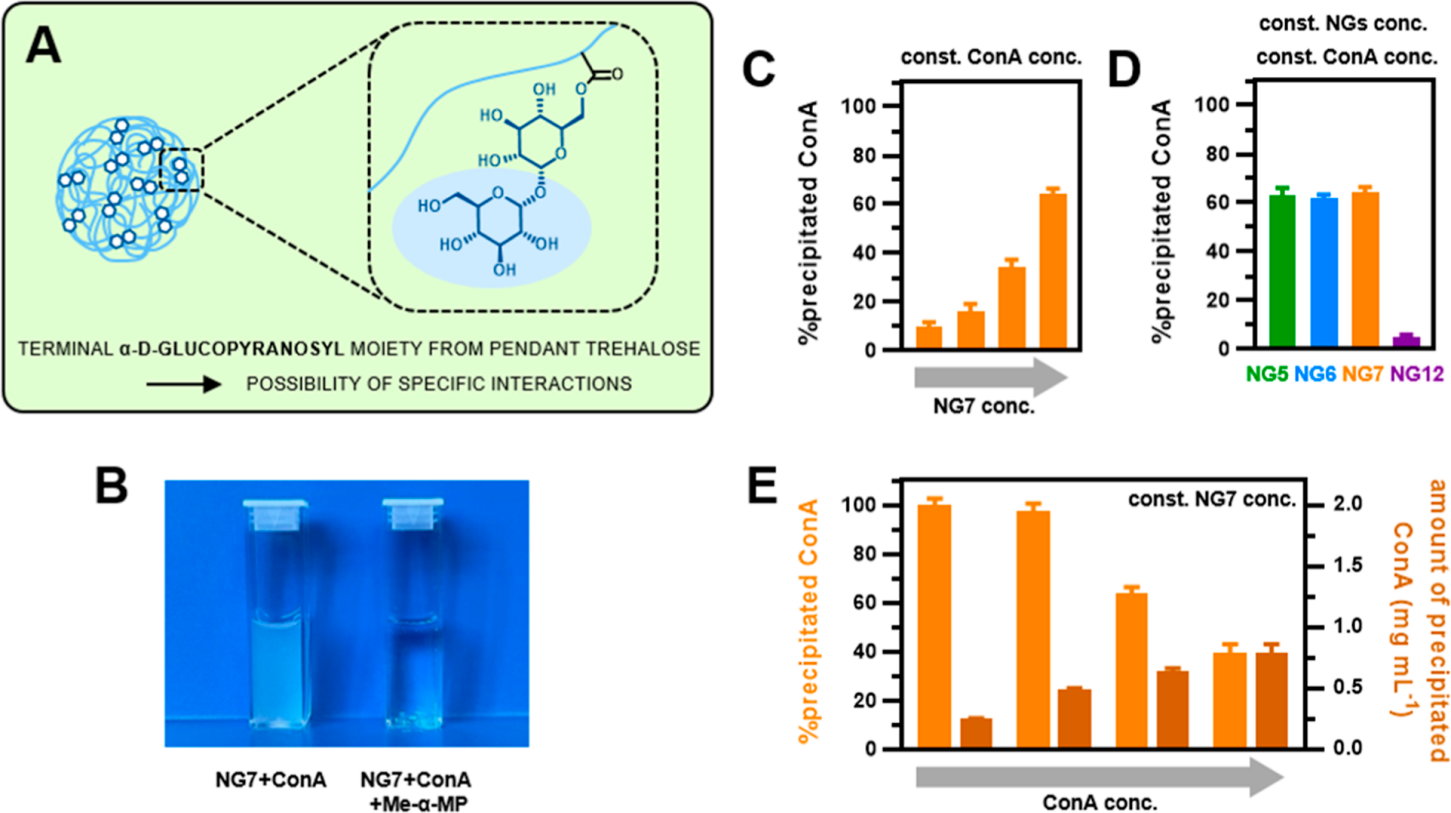
(A) Scheme highlighting the terminal α-d-glucopyranosyl moiety from pendant trehalose. (B) Photo of precipitate formation after adding 500 μM **NG7** nanogel dispersion into ConA solution (1.0 mg/mL) (left) and no precipitate formation after adding the same **NG7** nanogel dispersion into ConA solution containing 25 mM methyl-α-D-mannopyranoside (Me-α-MP) (right). (C) Quantitative precipitation of ConA at constant ConA concentration (1.0 mg/mL) and varying **NG7** nanogel concentration (**NG7** concentration increases from left to right as follows: 25, 50, 125, and 250 μM). (D) Quantitative precipitation of ConA by nanogels with various ionic functionalities (ConA: 1.0 mg/mL, nanogels: 250 μM). (E) Quantitative precipitation of ConA at constant **NG7** nanogel concentration and varying ConA concentration (ConA concentration increases from left to right as follows: 0.25, 0.5, 1.0, and 2.0 mg/mL). Nanogel concentration is given with respect to the concentration of the incorporated trehalose/sucrose.

Trehalose is composed of two α,α′–1,1′–linked glucopyranoses. Hence, its incorporation by using 6-*O*-monoacrylate gives nanogels with terminal α-d-glucopyranosyl moieties (Figure 5A). The decoration of nanocarriers with pendant glucose is frequently introduced to provide targeting capabilities or increase cellular uptake through specific interactions with glucose transporters (GLUTs). For example, glucosylated nanoparticles/polymers have been developed to improve tumor targeting owing to the overexpression of GLUTs in cancer cells^32,33^ or to facilitate BBB crossing and enhance brain accumulation via GLUT mediation.^34,35^

To preliminarily explore the biological availability of pendant α-d-glucopyranosyl moieties from trehalose attached to nanogels, their interactions with Concanavalin A (ConA) were examined. ConA is a lectin that exhibits specific affinity toward α-d-glucopyranosyl and α-d-mannopyranosyl residues, and it is widely used to gain first insights into the possibility of specific interactions of glycopolymers with such moieties.^36−38^ The interactions between nanogels and ConA were evaluated by quantitative precipitation assay for three nanogels differing in ionic functionality, **NG5**, **NG6**, and **NG7**, as well as for the sucrose-containing cationic nanogel **NG12**. At the assay pH (7.2), ConA exists as a tetramer with four binding sites, and thus, upon binding with a multivalent ligand, it forms cross-linked, insoluble complexes and precipitates. Indeed, trehalose-containing nanogels could precipitate ConA, which proved the binding between these two species (Figure 5B, left). The amount of precipitated ConA was positively correlated to the nanogel concentration (Figure 5C). Nanogels with cationic, anionic, or zwitterionic moieties all precipitated a similar amount of ConA (ca. 60%, Figure 5D), which indicated that α-d-glucopyranosyl moieties from trehalose on all these nanogels were equally accessible for interactions with ConA and that the binding was not influenced by the ionic functionality. In contrast, the sucrose-containing nanogel, **NG12**, precipitated less than 5% of the ConA. Sucrose was attached to the nanogel at its 6′ position, so it also introduced terminal α-d-glucopyranosyl moieties to the nanogel; however, the affinity of the α-d-glucopyranosyl moiety in sucrose/sucrose-containing glycopolymers toward ConA has been previously shown to be much lower than that from trehalose/trehalose-containing glycopolymers.^39^ Hence, the differences in the binding affinity of trehalose and sucrose in their respective nanogels toward ConA, exhibited as the difference in the amount of precipitated ConA, are a proof that the binding and precipitation of ConA are driven by the molecular recognition of α-d-glucopyranosyl moieties and not by other nonspecific interactions.

Another confirmation of these interactions’ specificity was obtained by assessing the precipitate formation in the presence of a large excess of the competitive monovalent ligand methyl-α-d-mannopyranoside, where none of the ConA precipitated (Figure 5B, right). In this case, methyl-α-d-mannopyranoside saturates binding sites on ConA, making them unavailable for specific interactions with nanogels. Also, the addition of a large amount of methyl-α-mannoside to the previously formed ConA-nanogel precipitate caused its dissolution due to the competitive binding with ConA and the displacement of the multivalent ligand. Finally, ConA precipitation at varying ConA concentrations and constant nanogel concentration was studied (using **NG7** as an example) (Figure 5E). The amount of precipitated ConA (expressed as a concentration) increased along with its concentration; however, the increase was not linearly proportional; it could be related to the progressive saturation of the nanogel, owing to the decreasing number of α-d-glucopyranosyl moieties available for binding interactions.

### Interactions of Nanogels with Serum

When considering the use of trehalose-releasing nanogels in nanomedicine, it is important to account for how these nanocarriers behave within the biological environment. In general, interactions between nanoparticles and their environment can lead to unpredictable changes in nanoparticle-related outcomes, including alterations in their function, uptake, biodistribution, immune responses, and toxicity.^40^ Particularly, it is widely recognized that when nanoparticles are introduced into biological fluids, they form a biological coating known as the biomolecular corona, which is composed of proteins, lipids, saccharides, nucleic acids, and metabolites on nanoparticle surfaces. The dominant effect is the direct adsorption of proteins from biological fluid onto nanoparticles, which depends on both the type of protein involved and the physicochemical characteristics of the nanoparticles, such as their size, hydrophilicity, and surface chemistry.

**Figure 6:**
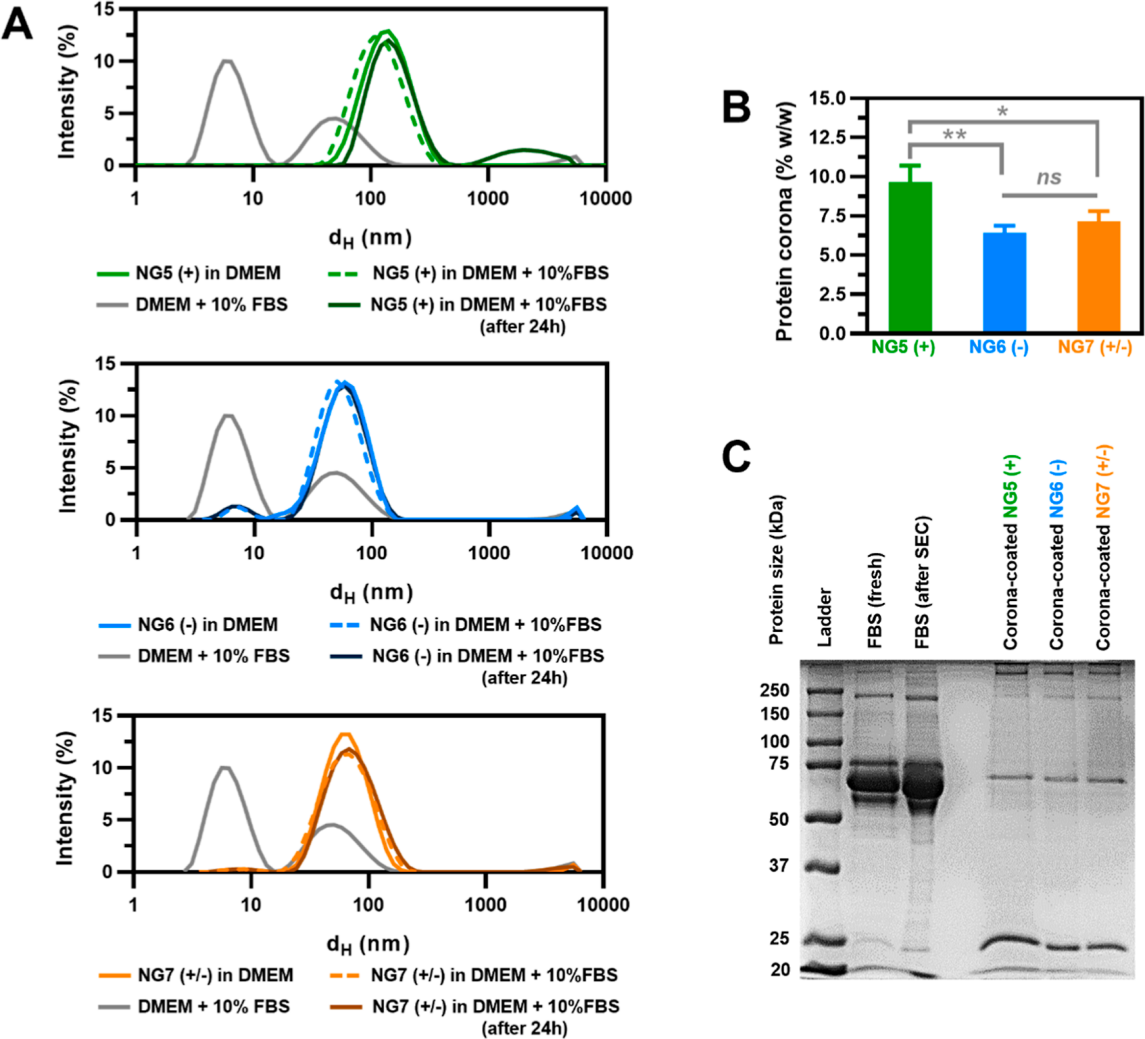
(A) DLS studies on colloidal stability of nanogels with cationic, anionic, or zwitterionic functionality: size distribution by DLS of 1.0 mg/mL **NG5** (+) (top), **NG6** (−) (middle), and **NG7** (±) (bottom) in DMEM with 10% FBS. (B) Quantification of protein corona adsorbed on nanogels (% w/w). Data are presented as mean ± SD (*n* = 3). Statistical analysis was performed using one-way ANOVA with Tukey’s Multiple Comparison. *p < 0.05, **p < 0.01, ***p < 0.001, ****p < 0.0001, and ^ns^*p* > 0.05 (not significant). (C) Visualization of protein corona from isolated corona-coated nanogels by SDS-PAGE. For FBS (fresh) and FBS (after SEC), ∼30 μg of protein was loaded, while for the corona-coated nanogels, ∼60 μg of nanogel was loaded.

We examined how the network charge of trehalose-releasing nanogels influenced their interactions with serum proteins. Three nanogels with different ionic functionalities, cationic (**NG5**), anionic (**NG6**), and zwitterionic (**NG7**), were chosen for studies on protein corona formation. Preliminary colloidal stability studies by DLS showed that all three nanogels were stable against aggregation in the cell medium supplemented with 10% FBS (Figure 6A). No substantial change in particle size via DLS, even after 24 h of incubation, was observed, suggesting that for this type of nanogel, protein binding may be lower than that observed for other types of materials (Figure 6A). Unfortunately, it was not possible to measure the ζ potential of nanogels in DMEM or in DMEM +10% FBS. An attempt to measure ζ potential of nanogels in DMEM has caused immediate electrode blackening (despite the adjusted voltage), rendering the measurement invalid. It is known that ζ potential measurements in cells with gold-plated electrodes are more challenging in complex buffer media with relatively high conductivity due to a number of factors, including electrode polarization, Joule heating, and electrode degradation. Therefore, to compare ζ potential before and after binding with serum proteins, the measurement was conducted as before in 1 mM KCl. In all cases, the binding of serum proteins on nanogels led to a reduction in the absolute value of their ζ potential. For nanogels with anionic and zwitterionic moieties, ζ potential changed from −17.6 to −12.2 mV and from −9.6 to −3.8 mV, respectively, while for cationic nanogel, it dropped from +30.2 to +14.3 mV.

To further confirm whether proteins were bound to the nanogels and to compare the corona composition for the nanogels with different ionic functionalities, fluorescent Cy5-labeled nanogels were incubated in full FBS. The corona-coated nanogels were separated from the excess serum proteins by size exclusion chromatography (SEC) using previously optimized procedures.^41^ The elution of corona-coated nanogels was monitored by measuring their fluorescence (at λ_ex_/λ_em_ = 640/681 nm) and the protein absorbance (at 280 nm) for each eluting fraction (Figure 6B, Figure S2). Quantification of the amount of proteins absorbed on the nanogels showed that the protein corona was formed in the range of 6–10% (w/w), with a slightly larger percentage in the case of the cationic nanogel. Zwitterionic polymers are known for their strong resistance to nonspecific protein adsorption. Herein, observed adsorption of proteins in the case of nanogel **NG7** containing zwitterionic moieties may result from its slightly negative network charge, which comes from the presence of carboxylate anions from the residual photoinitiator moieties.

SDS-PAGE gel electrophoresis was used for visualization of the relative distribution of the most abundant proteins in the recovered protein corona. The results suggested that neither the nanogel size nor the network charge seemed to substantially affect the composition of the protein corona. In fact, gel electrophoresis analysis of the isolated protein-coated nanogels showed a similar protein pattern for all the nanogels, with two major bands, one at ca. 25 kDa (along with a minor band of ca. 20 kDa), which may be assigned, based on molecular weight, to apolipoprotein A-I/II, and a second major band of ca. 65 kDa, which probably corresponded to serum albumin (Figure 6C). A comparable protein pattern and similar levels of serum protein adsorption on **NG5**, **NG6**, and **NG7** differing in network charges both suggest that for this type of nanogels, protein binding is influenced more by the chemical nature of the polymeric network (mainly consisting of acrylamide and trehalose acrylate units), rather than their network charge. Bewersdorff et al.^42^ observed that highly hydrophilic nanogels tend to interact preferably with apolipoproteins A and serum albumin. Trehalose-releasing nanogels are extremely hydrophilic, due to their high content of trehalose, and thus, the observed pattern of adsorbed bands may be consistent with such observations. Further studies, for instance, by mass spectrometry, are required to confirm the identity of the proteins in these bands and overall determine the composition of the adsorbed corona, as well as subtle changes for the nanogels of different charge.

### Internalization Studies of the Nanogels in HeLa Cells

**Figure 7:**
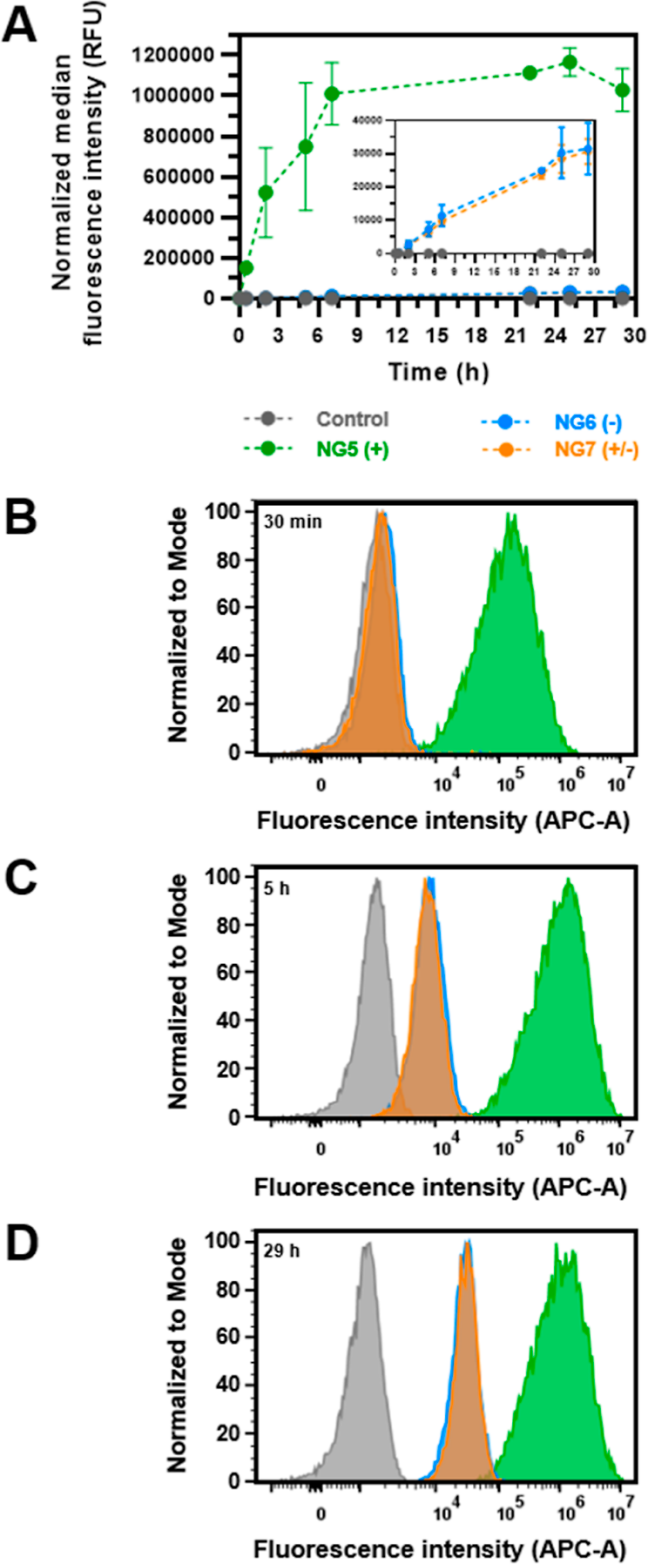
(A). Uptake kinetics of nanogels with cationic (**NG5**), anionic (**NG6**), and zwitterionic (**NG7**) functionality in HeLa cells for 29 h at 37 °C (nanogel concentration: 1 μg/mL). The inserted graph is the magnification of the lower fluorescence intensity range (0–40,000 RFU) showing the uptake of **NG6** and **NG7**. (B–D) Representative flow cytometry graphs of the fluorescence distribution of cells incubated with the nanogels for 30 min (B), 5 h (C), and 29 h (D) for **NG5** (green), **NG6** (blue), **NG7** (orange), and untreated cells (gray).

The next aim of the study was to correlate the charge of the nanogels with their ability to be internalized by HeLa cells. First, the cytotoxicity of nanogels against HeLa cells was examined. The results indicate that neither cationic (**NG5**), anionic (**NG6**), nor zwitterionic (**NG7**) nanogels caused cytotoxic effects influencing cell viability over the whole range of tested concentrations (10–1000 μg/mL) (Figure S3). The cellular uptake of Cy5-labeled nanogels was then studied by flow cytometry (Figure 7 and Figures S4–S7). Cationic nanogels were taken up to a significantly greater extent than the anionic or zwitterionic ones. The uptake kinetics showed that the cationic nanogels exposed to cells in MEM supplemented with 10% FBS had about 50 times greater uptake compared to anionic and zwitterionic nanogels (after 29 h of incubation) (Figure 7A). Furthermore, the uptake was significantly faster for cationic nanogels. A higher uptake for cationic nanoparticles is commonly observed, being usually attributed to the strong interaction with the negative charges of the cell surface. Cell membranes contain numerous negatively charged groups on their surface; hence, positively charged nanoparticles can interact more effectively through ionic interactions, which in turn promote their more effective endocytosis.^43^

**Figure 8:**
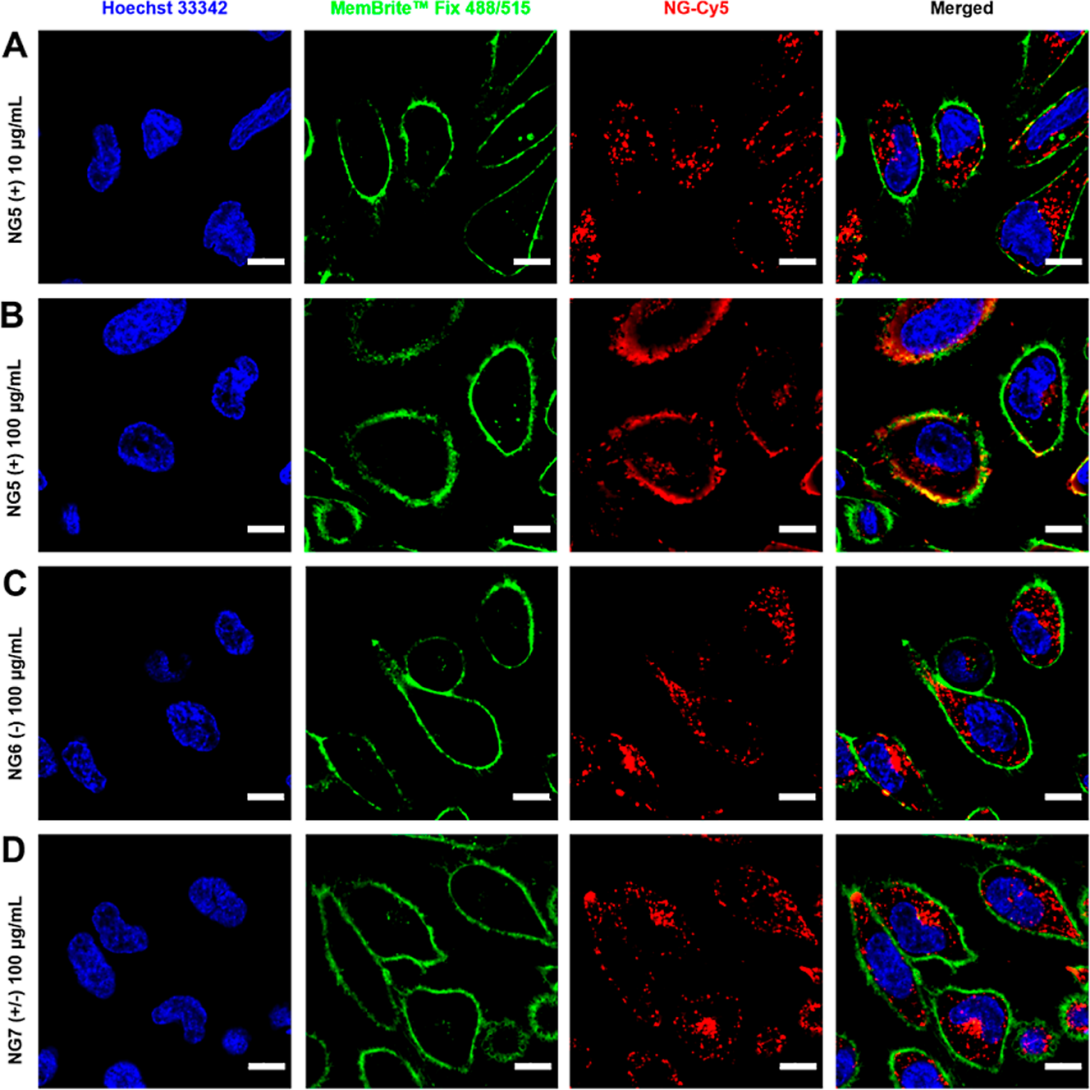
In vitro cell uptake of Cy5-labeled nanogels ((A) **NG5** (+) at 10 μg/mL, (B) **NG5** (+) at 100 μg/mL, (C) **NG6** (−) at 100 μg/mL, and (D) **NG7** (±) at 100 μg/mL) in HeLa cells after 5 h of incubation studied by confocal laser scanning microscopy. Nuclei are indicated in blue, membranes in green, and nanogels in red. (Scale bar = 10 μm.) Images depict representative cells selected from a larger population, which is presented in the ESI (Figures S8–S11).

The cellular uptake of differently charged nanogels was also studied by confocal microscopy, and the obtained images are shown in Figure 8 (the images depict representative cells selected from a larger population, which is shown in the Supporting Information (Figures S8–S11)). When incubated at a concentration of 100 μg/mL, some cationic nanogels were internalized by the cells, but the majority of them were found adhering on the cell membrane (Figure 8B). In contrast, anionic (Figure 8C) and zwitterionic (Figure 8D) nanogels when applied at the same concentration were internalized and distributed inside the cells, with no obvious differences observed between them. The uptake of cationic nanogels was more clearly visible when their concentration was diluted 10-fold. At a concentration of 10 μg/mL, cationic nanogels could be easily observed inside cells without the strong accumulation on the cell membrane, which was observed at a higher concentration (Figure 8A). In the meantime, at a reduced concentration of 10 μg/mL, the uptake of anionic and zwitterionic nanogels was poorly detectable in comparison. Overall, the results from microscopic imaging confirmed a significantly higher uptake capability of cationic nanogels compared to the other two. However, it is important to note that excessively high concentration of cationic nanogels can lead to their substantial accumulation on the cell membrane.

As a next step, various transport inhibitors were used to characterize the mechanisms of uptake involved in the internalization of the examined nanogels. Given their strongly higher uptake, for these studies, the concentration of the cationic nanogel was reduced 20-fold with respect to the other nanogels, in order to reduce the potential contribution of adhering nanoparticles to the cell fluorescence quantification by flow cytometry and ensure that the measured fluorescence would fit within the employed scale.

**Figure 9:**
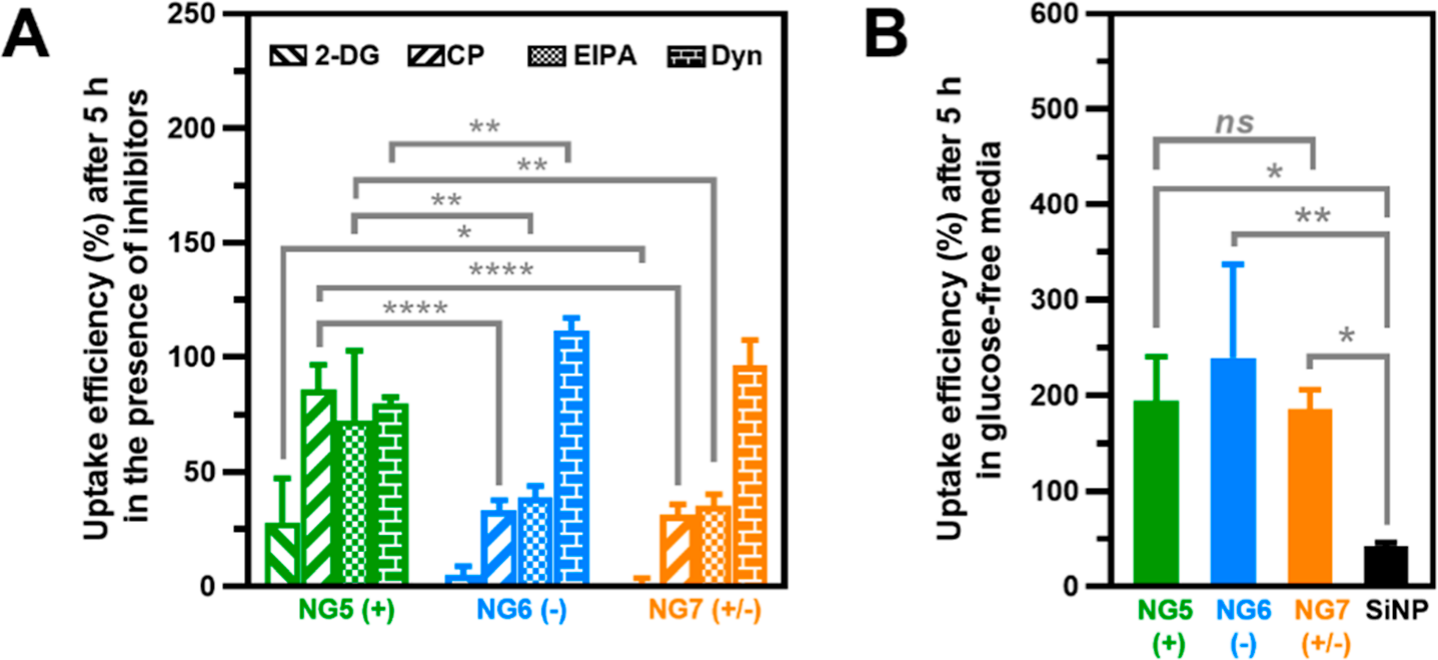
(A) Uptake efficiency of nanogels with cationic (**NG5**), anionic (**NG6**), and zwitterionic (**NG7**) functionality in HeLa cells at 5 h (37 °C) in the presence of different inhibitors: 2-deoxy-glucose (2-DG), chlorpromazine (CP), 5-(*N*-ethyl-*N*-isopropyl)-amiloride (EIPA), and hydroxy-dynasore (Dyn). Data are normalized for the uptake in cells in the absence of inhibitors. Statistical analysis was performed using two-way ANOVA with Tukey’s Multiple Comparison. *p < 0.05, **p < 0.01, ***p < 0.001, ****p < 0.0001, and ^ns^*p* > 0.05 (not significant). NG5 vs NG6 (2-DG)^ns^, NG5 vs NG7 (Dyn)^ns^, NG6 vs NG7 (2-DG, CP, EIPA, Dyn)^ns^. (B) Uptake efficiency of nanogels with cationic (**NG5**), anionic (**NG6**), and zwitterionic (**NG7**) functionality and SiNP in HeLa cells at 5 h (37 °C) assessed in glucose-free cell media. Data are normalized for the uptake in cells in media containing standard glucose concentration (1 g/L, ∼5.5 mM) (both media were supplemented with 10% FBS). Nanogel concentration: **NG5**: 0.1 μg/mL and **NG6** and **NG7**: 2 μg/mL). SiNP concentration: 25 μg/mL. Data are presented as mean ± SD (*n* = 3) and (*n* = 4) for **NG6** and SiNP on (B). Statistical analysis was performed using one-way ANOVA with Tukey’s Multiple Comparison. *p < 0.05, **p < 0.01, ***p < 0.001, ****p < 0.0001, and ^ns^*p* > 0.05 (not significant).

First, a glucose-free medium with 2-deoxy-glucose (2-DG) was used to deplete cell energy and determine whether nanogel uptake was energy-dependent. The results showed that under energy-depleted conditions, the uptake decreased substantially for all nanogels, confirming that all of them were taken up by means of energy-dependent mechanisms (Figure 9A).

The mechanism of endocytosis was then characterized by using various inhibitors of uptake pathways: chlorpromazine (CP) as a clathrin pathway inhibitor, 5-(*N*-ethyl-*N*-isopropyl)-amiloride (EIPA) as an inhibitor of macropinocytosis, and hydroxy-dynasore (Dyn) as an inhibitor of dynamin. Careful studies were previously performed to optimize the concentration to use on HeLa cells in order to confirm inhibition but exclude toxicity.^44^ The results showed that for the neutral and negative nanogels, endocytosis was significantly inhibited by both CP and EIPA (about 60% reduction), suggesting that these nanogels enter HeLa cells by a combination of these pathways. Instead, for the cationic nanogel, the decrease was lower and uptake was reduced only by 15–30% (Figure 9A). These results would suggest that clathrin-mediated endocytosis and macropinocytosis have minor roles in the uptake of these nanogels. Further studies should be performed to confirm this, for instance, using other methods and, given the very high uptake observed for the cationic nanogels, to fully exclude contamination of residual nanogels adhering outside of the cell membrane in the measured fluorescence.

In cells exposed to the dynamin inhibitor, surprisingly, for all nanogels, no reduction in uptake was observed. This result is in strong contrast with the uptake reduction observed with CP, which suggested the involvement of clathrin-mediated endocytosis in the uptake of the zwitterionic and negative nanogels. In fact, dynamin is involved in several endocytosis pathways, including clathrin-mediated endocytosis, where it assists the scission and release of intracellular endocytic vesicles.^45^ Based on the results observed with CP, one would expect that inhibiting dynamin-dependent processes would reduce nanogel uptake as well. However, it is known that blocking endocytosis may lead to the activation of compensatory mechanisms, confusing the interpretation of the results.^44,46,47^ For instance, given that incubation with EIPA had effects comparable to CP on uptake efficiency, the lack of effects with the dynamin inhibitor may be explained by an increased uptake by macropinocytosis as a compensatory mechanism. Further studies using different methods are required to confirm this possible interpretation and overall to test whether dynamin is involved in the uptake of the different nanogels. Overall, while further studies are required to fully identify the pathways involved in the uptake of the different nanogels, these preliminary results confirm that for the cationic nanogels, a different mechanism may be in place.

Another key component of many endocytic pathways, including clathrin-mediated endocytosis, is the interaction with receptors on the cell membrane. The trehalose-bearing nanogels can potentially interact with GLUTs through their terminal α-d-glucopyranosyl moieties from trehalose, which are pendant residues in the nanogel structure. The accessibility of these α-d-glucopyranosyl moieties for specific interactions was proven in the study with ConA lectin, described in the previous section. In order to test whether nanogel uptake was mediated by interaction with GLUTs, the nanogels were added to cells in a glucose-free medium. The significant increase (2–3 times) in uptake efficiency in the glucose-free medium in comparison to a medium containing standard glucose concentration (∼5.5 mM) may suggest that glucose competed with the nanogels for interactions with GLUTs and thus that interactions of the nanogels with GLUTs were involved in their uptake (Figure 9B). Under the same conditions, silica nanoparticles (SiNP), used as a control without glucose functionalization, exhibited a considerably reduced uptake (<50%), possibly due to additional effects of glucose depletion on the cell energy levels.

## Conclusions

Co-incorporation of trehalose (meth)acrylate together with hydrophilic primary/secondary acrylamides in one polymeric network resulted in hydrogels with covalent conjugation of trehalose, which was hydrolytically labile at physiologically relevant conditions. The utilization of photoinitiated FRP in a w/o miniemulsion for their synthesis was an effective way to reduce the size to the nanoscale and fabricate trehalose-releasing nanogels, which can potentially be used as trehalose nanocarriers for its sustained delivery. Furthermore, the selection of trehalose diacrylate as a cross-linker enabled the fabrication of nanogels that could undergo hydrolytical disintegration. The current approach also offers versatility in the selection of the ionic functionality of the nanogel (from among tertiary ammonium cation, quaternary ammonium cation, zwitterion (sulfobetaine), and carboxylate anion) through the simple selection of the relevant ionic acrylamide for their synthesis. The trehalose-releasing nanogels with optimized composition were characterized by an extremely high trehalose loading, which could constitute even more than 50% w/w of the weight of the nanogel and exhibited excellent colloidal stability in a serum-containing medium.

The current research gave an in-depth insight into the dependence of trehalose release on nanogel composition and showed that faster trehalose release is favored by a higher acrylamide to trehalose ratio; a higher primary to secondary amide ratio; incorporation of trehalose through acrylate rather than methacrylate; and incorporation of trehalose through monoacrylate rather than diacrylate. The trehalose release rate was also influenced to some extent by the ionic groups attached to the nanogel, decreasing in the following order for the studied functionalities: tertiary ammonium cation > quaternary ammonium cation ∼zwitterion (sulfobetaine) ≫ carboxylate anion. The hydrolysis-driven mechanism of trehalose release caused the release rate to be significantly dependent on the solution pH. It became faster as the pH increased, but it could still proceed even at the moderately acidic conditions of pH 6.5. Otherwise, the trehalose release rate was not dependent on concentration, which is very desirable for drug delivery because, in practice, it means that the release rate will not be influenced by the dilution, e.g., upon intravenous injection, and the amount of released trehalose will always be proportional to the amount of applied nanogel.

The nanogels exhibited a tendency for protein corona adsorption but without substantial differences with regard to both its amount and composition between nanogels with different ionic functionalities. Protein corona was quantified to form in the range of 6–10% w/w and 2 major bands were visible for all compositions, possibly coming from apolipoprotein A-I/II and serum albumin—as assigned based on their molecular weight. Further studies are required to confirm this, as well as to determine more subtle differences in composition, depending on nanogel charge. All three nanogels were taken up by HeLa cells following energy-dependent mechanisms; however, cationic nanogels were taken up much faster and to a significantly greater extent than the anionic or zwitterionic ones, consistent with the common observation of higher uptake for cationic nanoparticles. The results from preliminary studies on the mechanisms involved in the internalization of nanogels suggested that the uptake mechanism was likely to be different for nanogels with cationic moieties than for those with anionic and zwitterionic ones, but further studies are necessary to fully identify the exact pathways involved in the uptake of the differently charged nanogels. The results from confocal imaging confirmed the differences in the interactions with cells and the uptake of the cationic nanogel compared to the other two. Terminal α-d-glucopyranosyl moieties of the trehalose pendant from nanogels were accessible for molecular recognition by glucose binding units, as confirmed by the experiments with ConA. Consistent with these results, the greatly increased uptake of nanogels in a glucose-free medium suggested that interactions of nanogels with GLUT receptors mediated by d-glucopyranosyl moieties from pendant trehaloses may be involved in nanogel uptake. This study presents preliminary findings on some biologically relevant aspects and establishes a foundation for further biological research on trehalose-releasing nanogels. The follow-up studies should focus particularly on investigating the cellular performance of these nanocarriers, specifically examining intracellular trehalose release and biological effects of controlled trehalose delivery, as well as extend the research to other cell lines.

Overall, there is still a long way to confirm the biological activity, safety, and effectiveness of trehalose-releasing nanogels, but taking into account that currently there is no other strategy offering sustained release of trehalose at systemic pH from a nanocarrier to which trehalose is covalently conjugated, the presented results show that these nanogels represent a promising approach.

## Supporting Information Available

Monomer feed composition for nanogels synthesis; standard curves for determination of proteins and nanogels concentration; elution graphs from isolation of protein-coated nanogels by SEC; cytotoxicity data; additional data on uptake kinetics of nanogels; and additional images from in vitro cell uptake study by confocal laser scanning microscopy (PDF)
