# Supplementary material for: Trehalose-Releasing Nanogels: Study on Trehalose Release and Insights into Selected Biologically Relevant Aspects
Source: Biomacromolecules. 2025 Apr 14;26(5):2835–51. doi: 10.1021/acs.biomac.4c01505 (PMC12076505; doi:10.1021/acs.biomac.4c01505)
Supplement: Supplementary file 1 — bm4c01505_si_001.pdf [file bm4c01505_si_001.pdf]

Supplementary Information

for

Trehalose-releasing nanogels: study on trehalose release and insights into selected biologically relevant aspects

Ali Maruf [1,2], Małgorzata Milewska[1,2,*], Katarzyna Dudzisz[1,2,3], Anna Lalik[2,4], Sebastian Student[2,4], Anna Salvati[5], Ilona Wandzik[1,2]

[1] Department of Organic Chemistry, Bioorganic Chemistry and Biotechnology, Faculty of Chemistry, Silesian University of Technology, Krzywoustego 4, 44-100 Gliwice, Poland
[2] Biotechnology Center, Silesian University of Technology, Krzywoustego 8, 44-100 Gliwice, Poland
[3] Joint Doctoral School, Silesian University of Technology, Akademicka 2A, 44-100 Gliwice, Poland
[4] Department of Systems Biology and Engineering, Faculty of Automatic Control, Electronics and Computer Science, Silesian University of Technology, Akademicka 16, 44-100 Gliwice, Poland
[5] Department of Nanomedicine & Drug Targeting, Groningen Research Institute of Pharmacy, University of Groningen, A. Deusinglaan 1, 9713AV Groningen, The Netherlands

*Corresponding author: malgorzata.milewska@polsl.pl

Table S1. Monomer feed composition and yield of trehalose-releasing nanogels.

Nanogel	Trehalose monomer (mg, mmol)	Non-ionic monomer (mg, mmol)	Ionic monomer (mg, mmol)	Crosslinker (mg, mmol)	NHS-monomer (mg, mmol)	Yield (%)
**NG1**	TreA (80.6, 0.204)	-	AMPTMAC (125.0, 0.605)	MBAM (20.0, 0.130)	-	85
**NG2**	TreA (135.1, 0.341)	-	AMPTMAC (35.3, 0.171)	MBAM (20.0, 0.130)	-	74
**NG3**	TreA (163.1, 0.412)	-	AMPTMAC (17.6, 0.085)	MBAM (20.0, 0.130)	-	61
**NG4**	TreA (135.1, 0.341)	AM (35.3, 0.497)	AMPTMAC (35.3, 0.171)	MBAM (20.0, 0.130)	-	77
**NG5**	TreA (152.7, 0.385)	AM (35.3, 0.497)	AMPTMAC (17.6, 0.085)	MBAM (20.0, 0.130)	-	73
**NG6**	TreA (152.7, 0.385)	AM (35.3, 0.497)	AMBA (13.5, 0.085)	MBAM (20.0, 0.130)	-	67
**NG7**	TreA (152.7, 0.385)	AM (35.3, 0.497)	DAPS (23.7, 0.085)	MBAM (20.0, 0.130)	-	72
**NG8**	TreA (152.7, 0.385)	AM (35.3, 0.497)	DMAP (13.3, 0.085)	MBAM (20.0, 0.130)	-	65
**NG9**	TreMA (158.1, 0.385)	AM (35.3, 0.497)	AMPTMAC (17.6, 0.085)	MBAM (20.0, 0.130)	-	71
**NG10**	-	AM (35.3, 0.497)	AMPTMAC (17.6, 0.085)	TreDA (173.5, 0.385)	-	75
**NG11**	TreA (100.7, 0.254)	AM (35.3, 0.497)	AMPTMAC (17.6, 0.085)	TreDA (59.0, 0.131)	-	68
**NG12**	SucA* (152.7, 0.385)	AM (35.3, 0.497)	AMPTMAC (17.6, 0.085)	MBAM (20.0, 0.130)	-	74
**NG5-NHS**	TreA (152.7, 0.385)	AM (35.3, 0.497)	AMPTMAC (17.6, 0.085)	MBAM (20.0, 0.130)	AMBA-NHS (4.0, 0.011)	78[a]
**NG6-NHS**	TreA (152.7, 0.385)	AM (35.3, 0.497)	AMBA (13.5, 0.085)	MBAM (20.0, 0.130)	AMBA-NHS (4.0, 0.011)	92[a]
**NG7-NHS**	TreA (152.7, 0.385)	AM (35.3, 0.497)	DAPS (23.7, 0.085)	MBAM (20.0, 0.130)	AMBA-NHS (4.0, 0.011)	94[a]

*referes to sucrose instead of trehalose; [a]crude yield;

Standard curves of BSA and Cy5-labelled nanogels

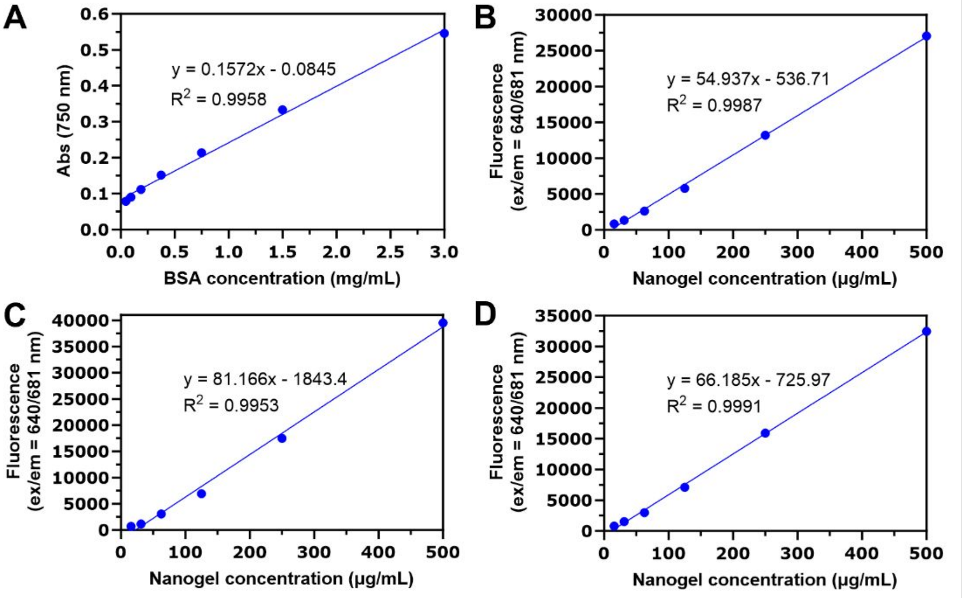

Figure S1. (A) Standard curve of BSA (0.047, 0.093, 0.188, 0.375, 0.75, 1.5, and 3 mg/mL). (B−D) Standard curve of Cy5-labelled nanogels (15.6, 31.3, 62.5, 125, 250, and 500 µg/mL), including (B) NG5-Cy5, (C) NG6-Cy5, and (D) NG7-Cy5.

Isolation of protein corona-coated nanogels

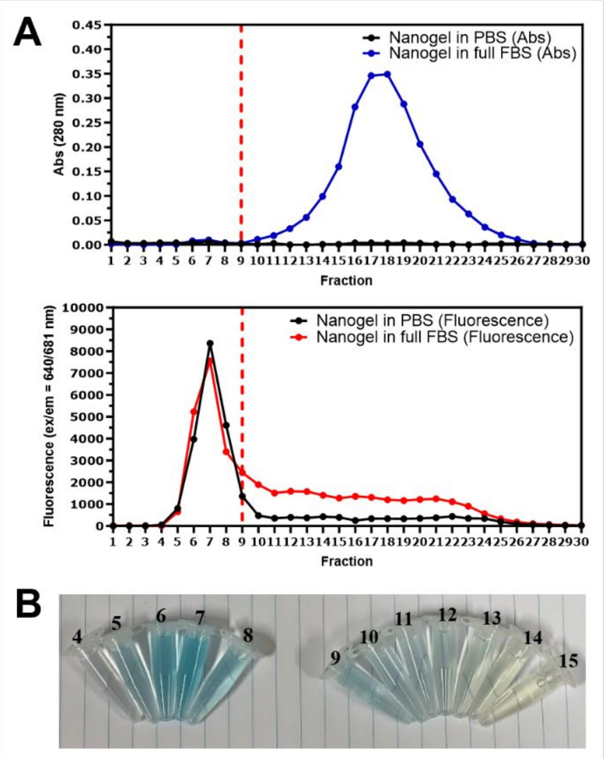

Figure S2. Isolation of protein corona-coated nanogels (A) A method to isolate corona-coated nanogels with Sepharose gel filtration chromatography. Fractions containing corona-coated nanogels were isolated after evaluating the absorbance (top) and fluorescence (bottom) of each fraction. (B) The appearance of collected fractions. Fractions 4-8 were selected for further analysis by gel electrophoresis for visualization of protein corona components.

Cytotoxicity profile of selected nanogels at different concentrations (10−1000 µg/mL) in HeLa cells for 24 h

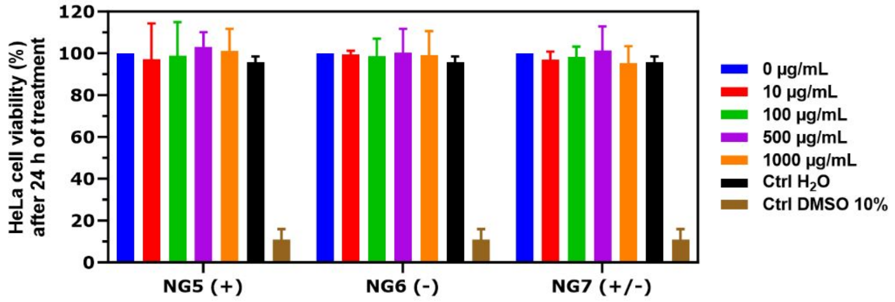

Figure S3. Cytotoxicity profile of selected nanogels: NG5 (+), NG6 (-), and NG7 (+/-) at different concentrations (10, 100, 500, and 1000 µg/mL) compared to control, control H_2O, and control DMSO 10% assessed in HeLa cells for 24 h of incubation at 37 °C. Data are presented as mean ± SD (*n* = 3).

Uptake kinetics and efficiency of Cy-5 labelled nanogels in the presence of 2-DG inhibitor

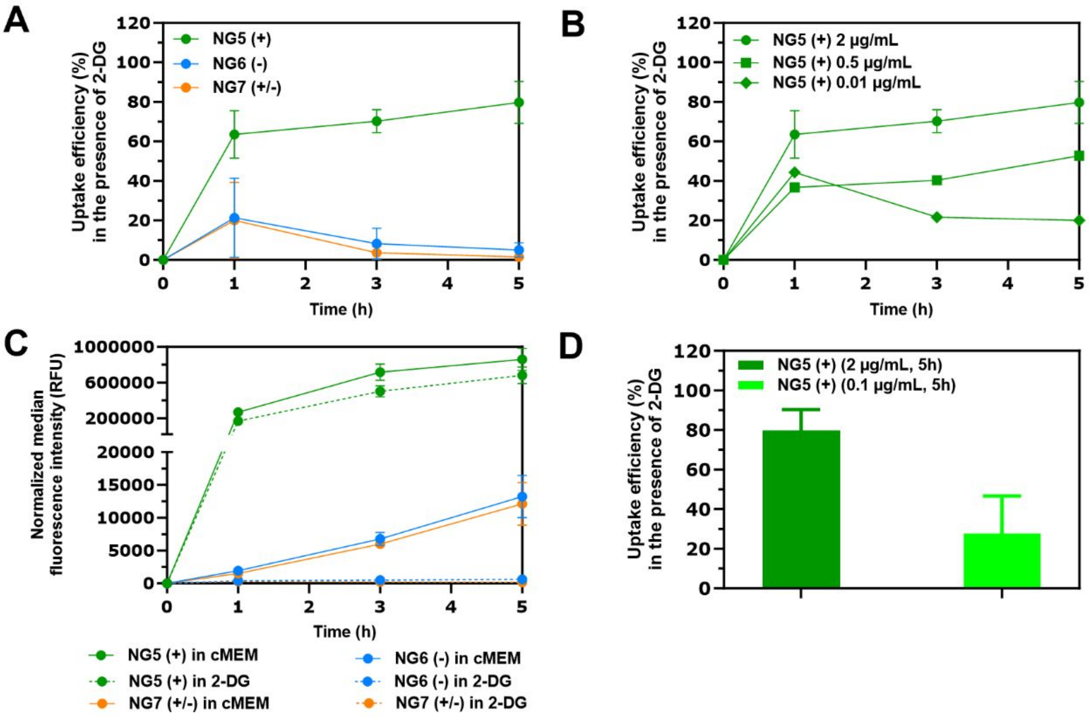

Figure S4. (A, C) Uptake kinetics of NG5 (+), NG6 (-), and NG7 (+/-) (at concentration: 2 µg/mL) over 5 h incubation with HeLa cells at 37 °C in the presence of 2-DG inhibitor. Data are presented as mean ± SD (*n* = 3). (B) Uptake kinetics of NG5 (+) at different concentrations (0.01, 0.5, and 2 µg/mL) over 5 h incubation with HeLa cells at 37 °C in the presence of 2-DG inhibitor. Data are presented as mean ± SD (*n* = 3), except concentrations of 0.01 and 0.5 µg/mL (*n* = 1, with 2 technical repeats). (D) Uptake efficiency of NG5 (+) at different concentrations (0.1 and 2 µg/mL) after 5 h incubation with HeLa cells at 37 °C in the presence of 2-DG inhibitor. Data are presented as mean ± SD (*n* = 3). The results show that for the positive nanogels, uptake levels were very high at 2 ug/ml and no effects was observed after incubation in energy depleted conditions with 2-DG. This was possibly due to the very high fluorescence levels and contribution from nanogels adhering outside the cells. Instead, when lowering the concentration to 0.1 ug/ml a strong inhibition was observed, confirming that uptake is energy dependent also for this nanogel.

Uptake kinetics and efficiency of Cy-5 labelled nanogels in the presence of CP inhibitor

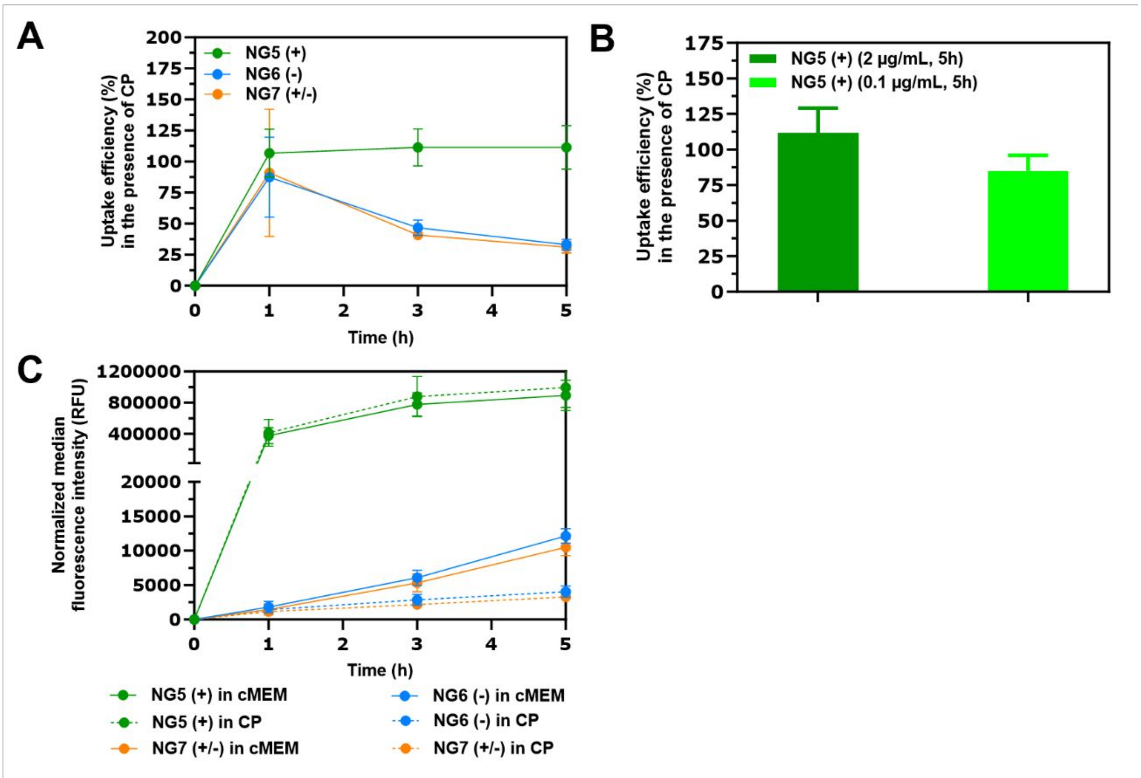

Figure S5. (A, C) Uptake kinetics of NG5 (+), NG6 (-), and NG7 (+/-) (at concentration: 2 µg/mL) over 5 h incubation with HeLa cells at 37 °C in the presence of CP inhibitor. (B) Uptake efficiency of NG5 (+) at different concentrations (0.1 and 2 µg/mL) after 5 h incubation with HeLa cells at 37 °C in the presence of CP inhibitor. Data are presented as mean ± SD (n = 3). The results show that uptake kinetics for NG6 (-) and NG7 (+/-) were significantly inhibited by CP over time suggesting the involvement of clathrin-mediated endocytosis. Meanwhile, NG5 (+) did not show any inhibition over time at 2 µg/mL but it showed slightly inhibition when lowering the concentration to 0.1 µg/mL, suggesting minor involvement of clathrin-mediated endocytosis.

Uptake kinetics and efficiency of Cy-5 labelled nanogels in the presence of EIPA inhibitor

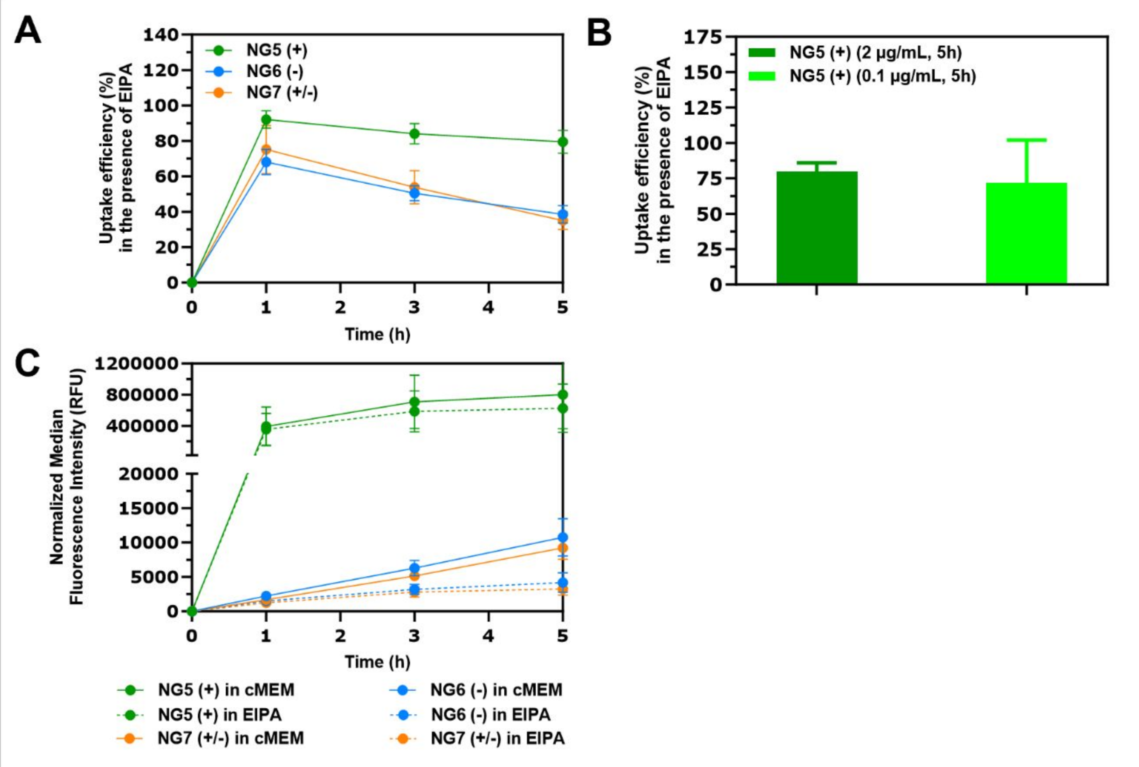

Figure S6. (A, C) Uptake kinetics of NG5 (+), NG6 (-), and NG7 (+/-) (at concentration: 2 µg/mL) over 5 h incubation with HeLa cells at 37 °C in the presence of EIPA inhibitor. (B) Uptake efficiency of NG5 (+) at different concentrations (0.1 and 2 µg/mL) after 5 h incubation with HeLa cells at 37 °C in the presence of EIPA inhibitor. Data are presented as mean ± SD (*n* = 3). The results showed that uptake kinetics for NG6 (-) and NG7 (+/-) were significantly inhibited by EIPA over time suggesting the involvement of macropinocytosis. Meanwhile, NG5 (+) showed minimal inhibition over time at 2 µg/mL as well as at 0.1 µg/mL, suggesting minor involvement of macropinocytosis.

Uptake kinetics and efficiency of Cy-5 labelled nanogels in the presence of Dyn inhibitor

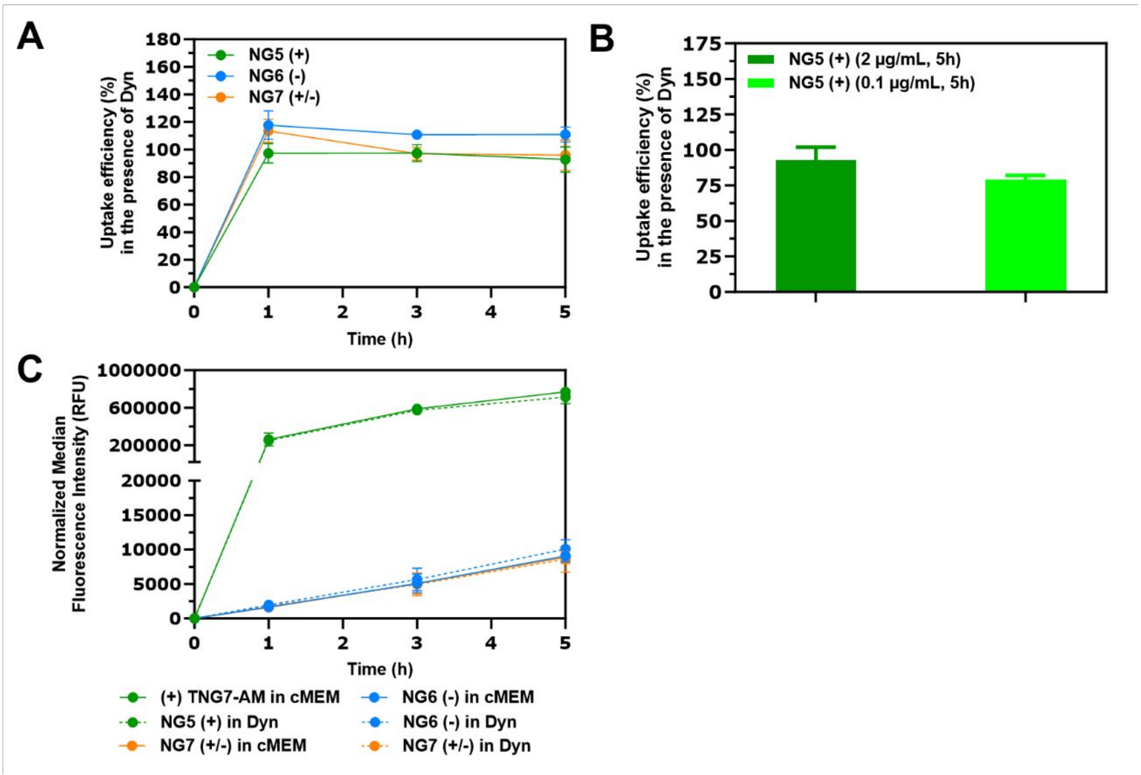

Figure S7. (A, C) Uptake kinetics of NG5 (+), NG6 (-), and NG7 (+/-) (at concentration: 2 μg/mL) over 5 h incubation with HeLa cells at 37 °C in the presence of Dyn inhibitor. (B) Uptake efficiency of NG5 (+) at different concentrations (0.1 and 2 μg/mL) after 5 h incubation with HeLa cells at 37 °C in the presence of Dyn inhibitor. Data are presented as mean ± SD (n = 3). The results showed that uptake kinetics for NG6 (-) and NG7 (+/-) were not inhibited by Dyn over time suggesting that there is possibly no involvement of dynamin on clathrin-mediated endocytosis or macropinocytosis. Meanwhile, NG5 (+) showed minimal inhibition over time at 2 μg/mL as well as at 0.1 μg/mL, suggesting minor involvement of dynamin on clathrin-mediated endocytosis or macropinocytosis. Further studies are necessary for confirming the influence of dynamin, possibly using different dynamin inhibitors.

Cell uptake of Cy5-labelled NG5 (+) at 10 ug/mL in HeLa cells

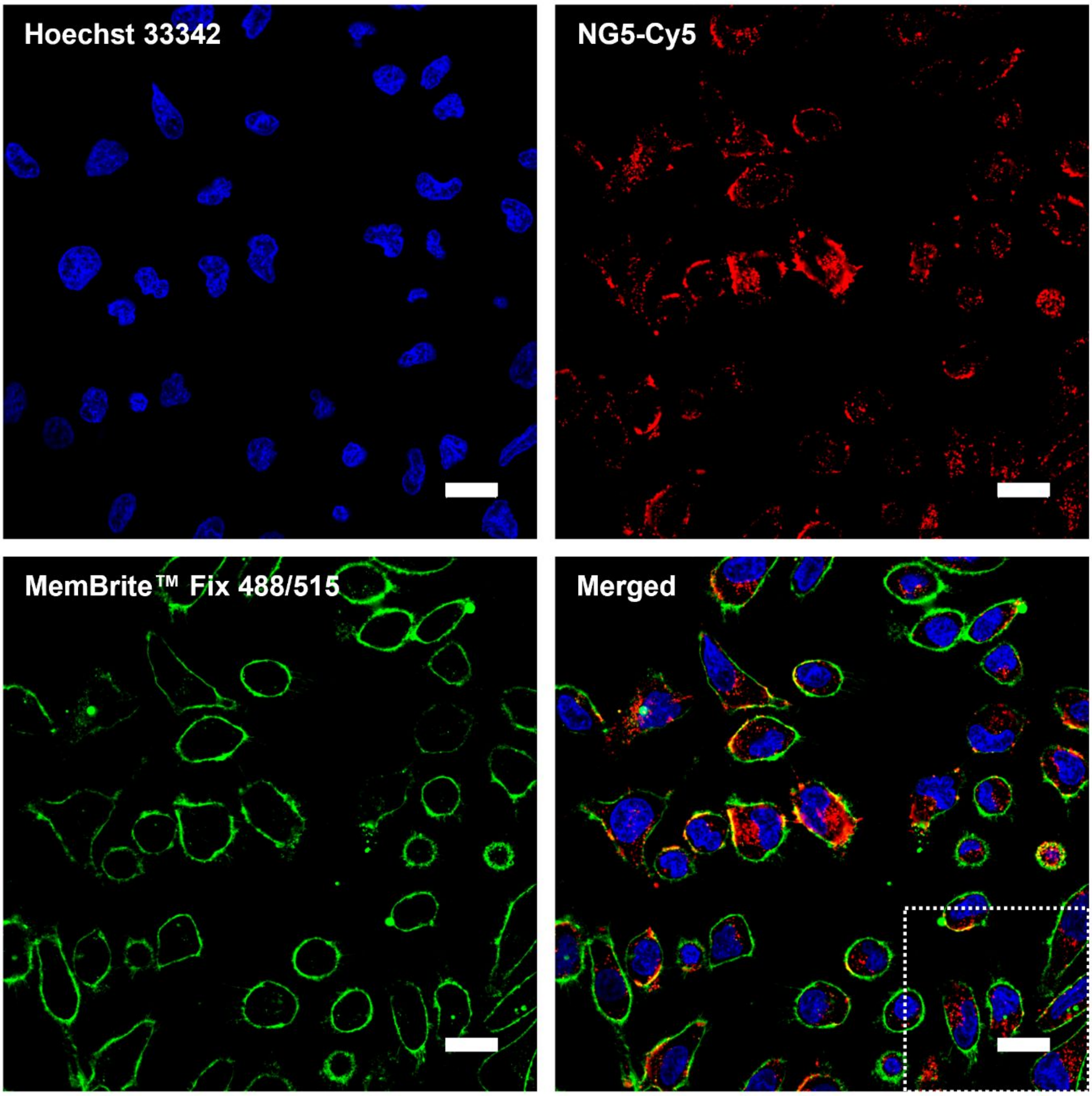

Figure S8. In vitro cell uptake of Cy5-labelled NG5 (+) (at 10 μg/mL in HeLa cells after 5 h of incubation) studied by confocal laser scaning microscopy. Nuclei are indicated in blue, membranes in green and nanogels in red. (Scale bars = 20 μm). Cells within the framed area were selected as representative and are presented at higher magnification in Fig. 8 of the main text.

Cell uptake of Cy5-labelled NG5 (+) at 100 ug/mL in HeLa cells

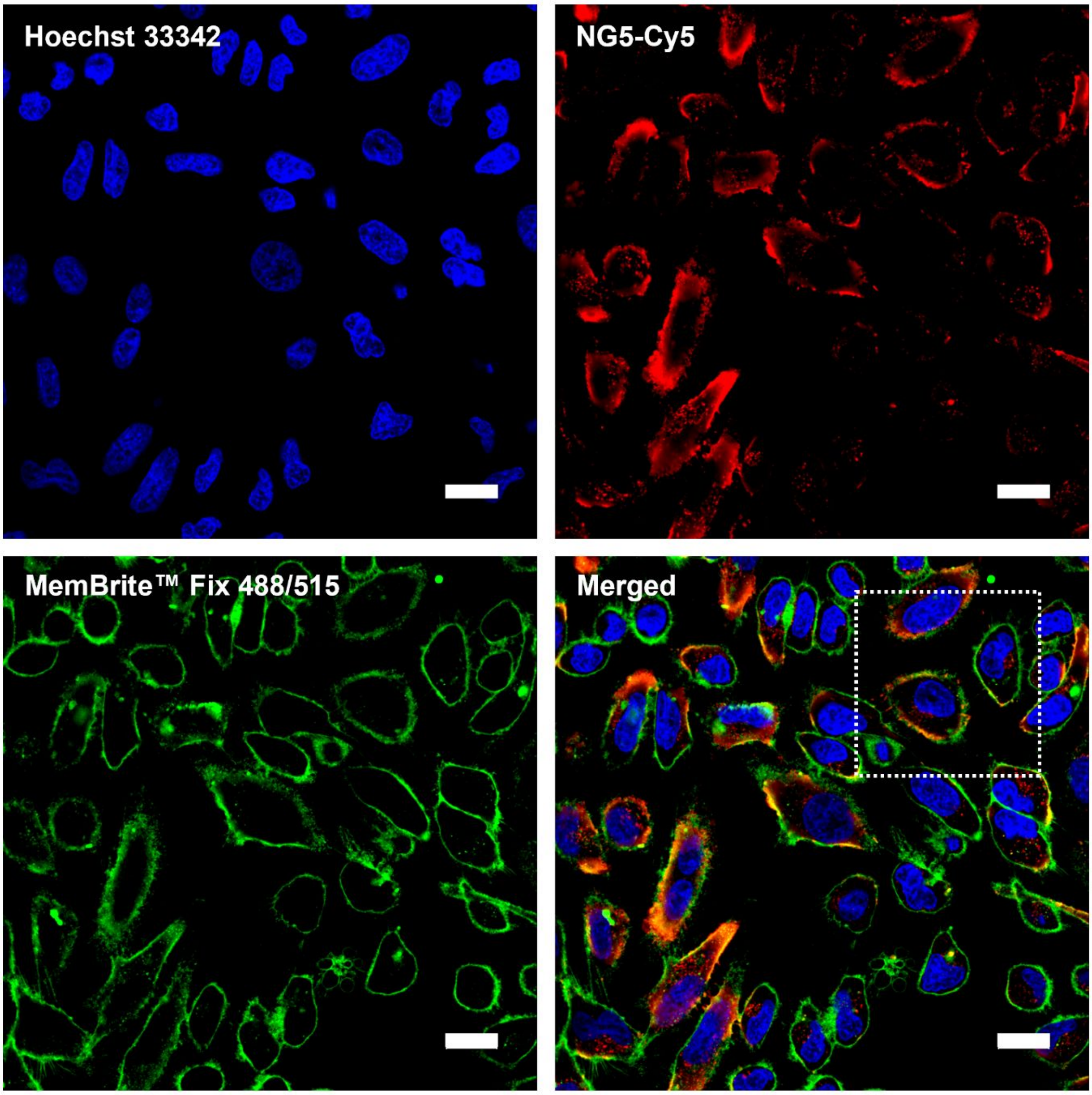

Figure S9. In vitro cell uptake of Cy5-labelled NG5 (+) (at 100 µg/mL in HeLa cells after 5 h of incubation) studied by confocal laser scaning microscopy. Nuclei are indicated in blue, membranes in green and nanogels in red. (Scale bars = 20 µm). Cells within the framed area were selected as representative and are presented at higher magnification in Fig. 8 of the main text.

Cell uptake of Cy5-labelled NG6 (-) at 100 ug/mL in HeLa cells

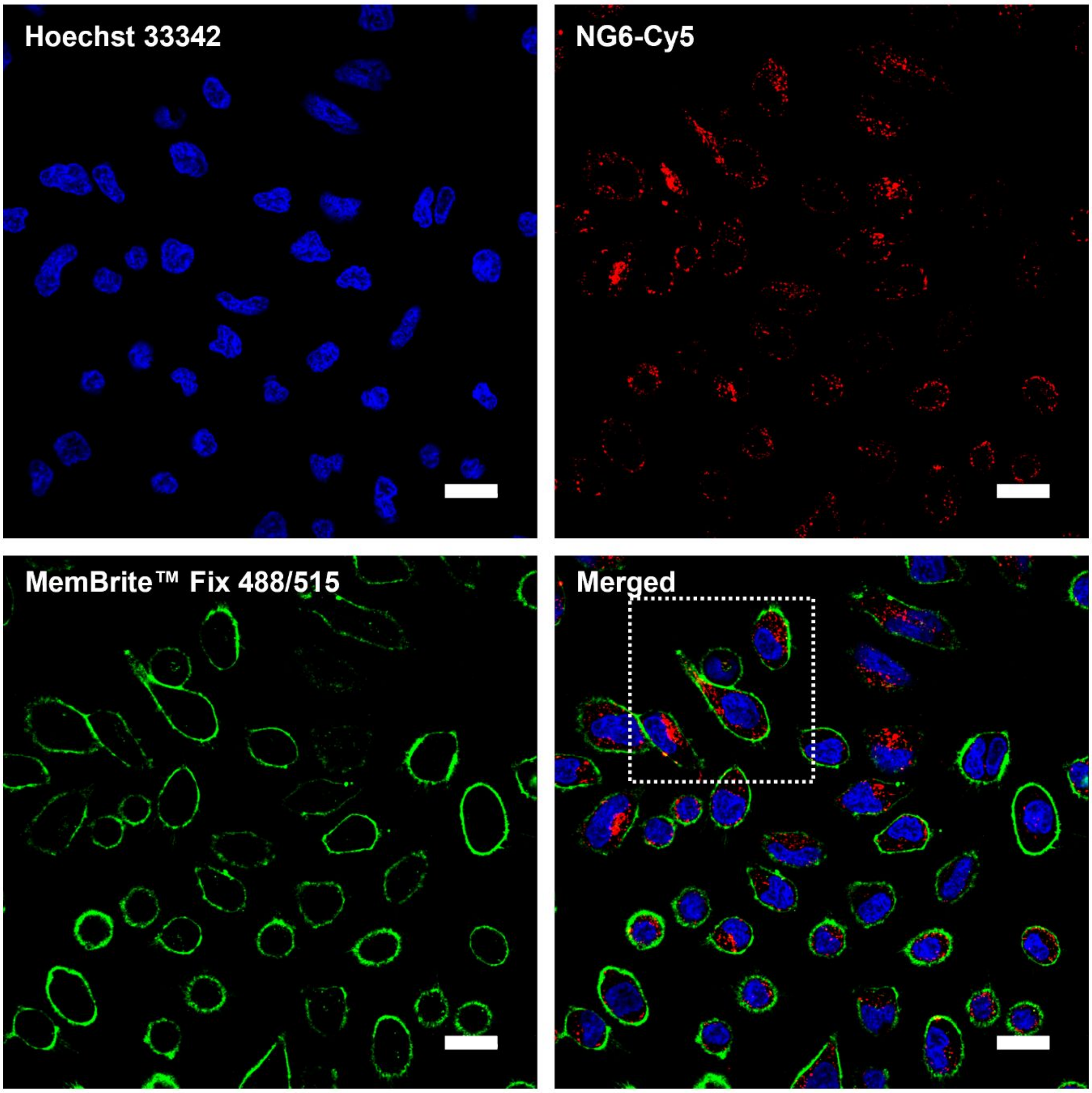

Figure S10. In vitro cell uptake of Cy5-labelled NG6 (-) (at 100 µg/mL in HeLa cells after 5 h of incubation) studied by confocal laser scaning microscopy. Nuclei are indicated in blue, membranes in green and nanogels in red. (Scale bars = 20 µm). Cells within the framed area were selected as representative and are presented at higher magnification in Fig. 8 of the main text.

Cell uptake of Cy5-labelled NG7 (+/-) at 100 ug/mL in HeLa cells

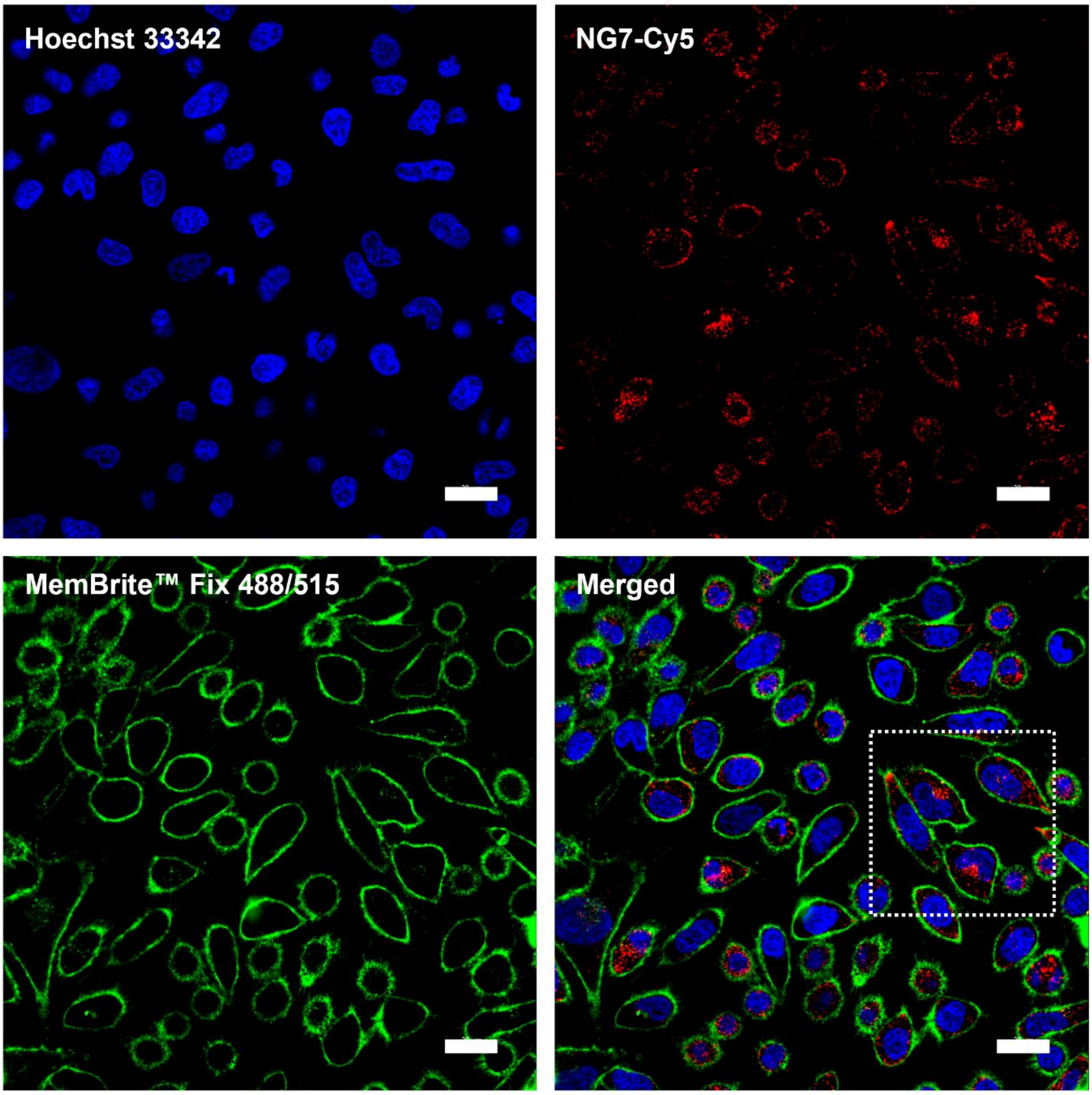

Figure S11. In vitro cell uptake of Cy5-labelled NG7 (+/-) (at 100 µg/mL in HeLa cells after 5 h of incubation) studied by confocal laser scaning microscopy. Nuclei are indicated in blue, membranes in green and nanogels in red. (Scale bars = 20 µm). Cells within the framed area were selected as representative and are presented at higher magnification in Fig. 8 of the main text.
